# Management of pulmonary thromboembolism in children: an evidence-based expert consensus

**DOI:** 10.1007/s12519-025-00987-3

**Published:** 2026-02-20

**Authors:** Li-Nan Zeng, Ying-Xue Zou, Hai-Lin Zhang, Li-Na Chen, De-Hui Chen, Xin-Xin Chen, Xing Chen, Zhi-Min Chen, Xiao-Yan Dong, Liang Huang, Yi Ji, Yong-Mei Jiang, Zhi-Ping Li, En-Mei Liu, Shu-Hua Luo, Xiao-Feng Ni, Guang-Min Nong, Yun Peng, Su-Yun Qian, Tian-You Wang, Xin-Yu Yuan, Hao Zhang, Hong Zhang, Xiao-Bo Zhang, De-Yu Zhao, Shun-Ying Zhao, Xiu-Fang Zhao, Kai-Yu Zhou, Quan Lu, Ling-Li Zhang, Han-Min Liu

**Affiliations:** 1 Department of Pharmacy/Evidence-Based Pharmacy Center, West China Second University Hospital, Sichuan University; Children’s Medicine Key Laboratory of Sichuan Province, Chengdu, China; 2 NMPA Key Laboratory for Technical Research on Drug Products In Vitro and In Vivo Correlation, Chengdu, China; 3 Key Laboratory of Birth Defects and Related Diseases of Women and Children, Sichuan University, Ministry of Education, Chengdu, China; 4 West China Biomedical Big Data Center, West China Hospital, Sichuan University, Chengdu, China; 5 Department of Pulmonology, Tianjin Children’s Hospital (Children’s Hospital of Tianjin University), Tianjin, China; 6 Department of Pediatric Respiratory Medicine, The Second Affiliated Hospital and Yuying Children’s Hospital of Wenzhou Medical University, Wenzhou, China; 7 Department of Pediatrics, West China Second University Hospital, Sichuan University, Chengdu, China; 8 Department of Pediatrics, The First Affiliated Hospital of Guangzhou Medical University, Guangzhou, China; 9 Heart Center, Guangdong Provincial Key Laboratory of Research in Structural Birth Defect Disease, Guangzhou Women and Children’s Medical Center, Guangzhou Medical University, Guangzhou, China; 10 Department of Pediatrics, Shandong Provincial Hospital Affiliated to Shandong First Medical University, Jinan, China; 11 Department of Pulmonology, Children’s Hospital, Zhejiang University School of Medicine, National Clinical Research Center for Child Health, Hangzhou, China; 12 Department of Pulmonology, Shanghai Children’s Hospital, School of Medicine, Shanghai Jiao Tong University, Shanghai, China; 13 Department of Pediatric Surgery, West China Hospital, Sichuan University, Chengdu, China; 14 Department of Laboratory Medicine, West China Second University Hospital, Sichuan University, Chengdu, China; 15 Department of Clinical Pharmacy, National Children’s Medical Center, Children’s Hospital of Fudan University, Shanghai, China; 16 Department of Respiratory Medicine, Children’s Hospital of Chongqing Medical University, Chongqing, China; 17 Department of Pediatrics, The First Affiliated Hospital of Guangxi Medical University, Nanning, China; 18 Department of Radiology, Beijing Children’s Hospital, Capital Medical University, National Center for Children’s Health, Beijing, China; 19 Pediatric Intensive Care Unit, Beijing Children’s Hospital, Capital Medical University, Beijing, China; 20 Hematology Center, Beijing Children’s Hospital, Capital Medical University, National Center for Children’s Health, Beijing, China; 21 Department of Radiology, The Affiliated Children’s Hospital, Capital Institute of Pediatrics, Beijing, China; 22 Heart Center and Shanghai Institute of Pediatric Congenital Heart Disease, Shanghai Children’s Medical Center, National Children’s Medical Center, Shanghai Jiaotong University School of Medicine, Shanghai, China; 23 Department of Clinical Laboratory, Shanghai Children’s Hospital, School of Medicine, Shanghai Jiao Tong University, Shanghai, China; 24 Department of Respiratory Medicine, Children’s Hospital of Fudan University, Shanghai, China; 25 Department of Respiratory Medicine, Children’s Hospital of Nanjing Medical University, Nanjing, China; 26 Department of Respiratory Disease, Beijing Children’s Hospital, Capital Medical University, Beijing, China; 27 Department of Nursing, West China Second University Hospital, Sichuan University/West China School of Nursing, Sichuan University, Chengdu, China; 28 Chinese Evidence-Based Medicine Center, West China Hospital, Sichuan University, Chengdu, China; 29 Department of Pediatric Pulmonology and Immunology, West China Second University Hospital, Sichuan University, Chengdu, China; 30 NHC Key Laboratory of Chronobiology (Sichuan University), Chengdu, China; 31 The Joint Laboratory for Lung Development and Related Diseases of West China Second University Hospital, Sichuan University and School of Life Sciences of Fudan University, West China Institute of Women and Children’s Health, West China Second University Hospital, Sichuan University, Chengdu, China; 32 Sichuan Birth Defects Clinical Research Center, West China Second University Hospital, Sichuan University, Chengdu, China

**Keywords:** Children, Diagnosis, Expert consensus, Management, Pulmonary thromboembolism, Treatment

## Abstract

**Background:**

Pulmonary thromboembolism is rare in children, but can be life-threatening. Timely diagnosis and treatment of pulmonary thromboembolism are crucial for reducing mortality associated with pulmonary thromboembolism in children. While guidelines for pulmonary thromboembolism in adults are available, guidelines for standardized diagnosis and management of pulmonary thromboembolism in children are not. This expert consensus aims to provide recommendations for the management of pulmonary thromboembolism in children based on the current best available evidence.

**Data sources:**

Following the World Health Organization Handbook for Guideline Development, the expert panel consisted of 30 members from different clinical areas. The panel identified clinical questions through systematic reviews and expert discussions, systematically reviewed evidence on pulmonary thromboembolism in children, and evaluated the quality of the evidence using the Grading of Recommendations Assessment, Development, and Evaluation (GRADE) approach. Using the GRADE Evidence to Decision Framework, the panel made recommendations, considering the effects of interventions, resource use, values and preferences, equity, acceptability, and feasibility.

**Results:**

The epidemiology, classification, and pathophysiology characteristics are summarized. The expert panel developed 33 recommendations addressing 20 questions related to diagnosis steps, treatment approaches such as anticoagulant therapy, thrombolysis therapy, catheter-based interventional therapy, surgical embolectomy, multidisciplinary team, and treatment of patients with comorbidities, prognosis, education, as well as follow-up. Among these, 18 are weak recommendations based on very low quality evidence, and 15 are good practice statements.

**Conclusions:**

The expert panel provided recommendations for pulmonary thromboembolism in children based on available evidence, which was generally low in quality and volume. The panel urges further research on early identification and diagnosis strategies, preventive and therapeutic regimens, and long-term management for pulmonary thromboembolism.

**Graphical abstract:**

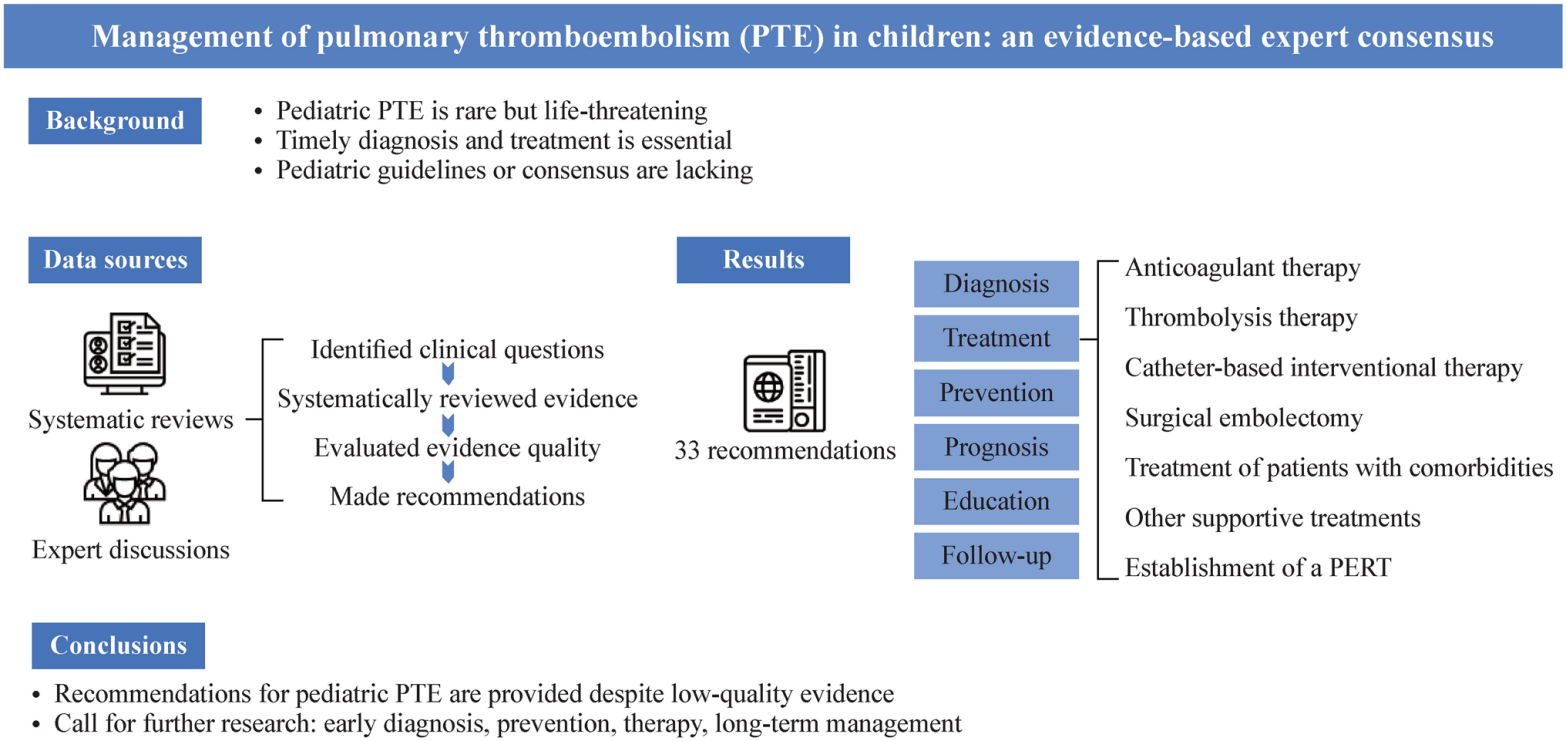

**Supplementary Information:**

The online version contains supplementary material available at 10.1007/s12519-025-00987-3.

## Introduction

Pulmonary thromboembolism (PTE) is a cardiovascular condition caused by a thrombus obstructing the pulmonary artery or its branches [1]. PTE is rare in children, but can be life-threatening. Timely diagnosis and treatment of PTE are crucial for reducing mortality associated with PTE in children [2]. While guidelines for PTE in adults are available [3, 4], guidelines for the standardized diagnosis and management of PTE in children are not. This expert consensus aims to provide recommendations for the diagnosis and management (including treatment, assessment of prognosis, patient education, and follow-up) of children (< 18 years of age) with PTE.

### Data sources

The expert panel is composed of 30 members including content experts in pediatric respiratory, infection, hematology, critical care, cardiology, surgery, laboratory medicine, medical imaging, nursing, pharmacy, and methodologists (Supplementary material 1). We registered this evidence-based expert consensus in the Practice guideline REgistration for transPAREncy registry (registration number: PREPARE-2023CN922). The development and report followed the World Health Organization Handbook for Guideline Development [5], the Appraisal of Guidelines for Research and Evaluation II [6] and the Reporting Items for Practice Guidelines in Healthcare [7].

Through a systematic search of existing guidelines, systematic reviews, and original studies related to PTE in adults and children along with formal discussions, the expert panel identified 20 clinical questions. The systematic review team conducted systematic reviews to address these clinical questions and will report the detailed methodology and results in separate publications [8, 9]. The expert panel conducted an online consultation questionnaire to assess patient preferences regarding specific interventions, integrating insights from clinical practice experience.

Following the Grading of Recommendations Assessment, Development and Evaluation (GRADE) Evidence to Decision (EtD) framework, the expert panel formulated recommendations. The panel categorized the strength of recommendations as strong and weak [10]. In the absence of evidence from current studies and based on experience from the expert panel, good practice statements were developed [11].

## Overview of pulmonary thromboembolism

### Definition

Pulmonary embolism (PE) refers to a group of diseases or clinical syndromes characterized by obstruction of the pulmonary artery or its branches by various types of emboli [1]. PTE is the most common type of acute PE, caused by thrombosis originating from the venous system, the right heart chambers, or in situ within the pulmonary arterial system; this leads to obstruction of the pulmonary artery or its branches. The main pathophysiological features and clinical manifestations of PTE are pulmonary circulatory and respiratory dysfunction [12]. PTE accounts for more than 90% of PE [13]. Non-thrombotic PE is defined as the partial or total occlusion of the pulmonary circulation caused by different non-thrombotic embolic agents. These include cells (such as adipocytes, hematopoietic, amniotic, trophoblastic, or tumor cells), bacteria, fungi, parasites, foreign material, or gas [14]. PTE and deep venous thromboembolism (DVT) are collectively referred to as venous thromboembolism (VTE), as they share the same predisposing factors and represent two clinical manifestations of VTE at different sites and stages [1].

### Epidemiology

Studies of children with PTE in the United States reported a prevalence of about 23 per 100,000 [15, 16], with two peaks occurring < 1 year old and > 15 years old [17–19]. Studies from the United States, Korea, and England revealed an increasing trend of PTE in children over the past decades [2, 20, 21]. The mortality rate of children with PTE was about 11.7% on average but with large variations between different countries [15, 16, 18, 22–24].

Risk factors for PTE in children can be categorized into hereditary and acquired. Hereditary factors include family history of VTE [23], specific gene mutations (i.e., *V Leiden* G1691A and G20210A gene mutations), congenital heart disease, elevated levels of human coagulation factors VIII/IX, protein C deficiency, protein S deficiency, antithrombin deficiency, hyperhomocysteinemia, and positive for antiphospholipid antibodies [25]. Acquired factors include immobility, surgery, central or deep vein catheterization, history of VTE, obesity, heart disease, tumor, trauma, and infection. Some additional potential risk factors include a hypercoagulable state, pulmonary disease, vascular malformation, congestive heart failure, use of psychotropic medications, use of oral contraceptives [26], chemotherapy, shunted hydrocephalus, dehydration, shock [27], nephrotic syndrome [24] and dermatomyositis [19].

### Classification

Based on classification criteria of PTE used in adults coupled with the experience of diagnosing PTE in children, the expert panel recommended the following classification for PTE in children: (1) based on time of onset, PTE can be categorized into acute and chronic PTE. Acute PTE is mainly characterized by the sudden onset of pulmonary hypertension with hypoxemia, typically lasting no longer than six months [3]. Suspected acute PTE or acute exacerbation of chronic PTE is when pulmonary arterial pressure exceeds 40 mmHg [3]. Chronic PTE is defined as abnormal pulmonary blood flow distribution and pulmonary circulation hemodynamics that persist for ≥ 6 months without substantial improvement [28]. (2) Based on thrombus source, PTE can be categorized as PTE from the inferior vena cava pathway, PTE from the superior vena cava pathway, PTE from the right heart cavity, and PTE from the in-situ thrombus [12]. Most thrombi originate from deep veins of the lower extremities; thrombus from the right heart cavity or in-situ thrombus accounted for a smaller proportion. However, thrombi from the superior vena cava pathway have increased. This is mainly related to an increase in intraventricular chemotherapy in the internal jugular vein and subclavian vein catheterization, peripherally inserted central venous catheters, or medium- and long-term catheters for intravenous chemotherapy [12]. (3) Based on pathogenesis, PTE can be categorized as idiopathic, secondary, and recurrent. (4) Based on the risk of pulmonary circulatory and respiratory dysfunction, PTE can be categorized as high-risk PTE, intermediate-risk PTE, and low-risk PTE [29, 30] (Table 1).

**Table 1:** Risk classification of children with pulmonary thromboembolism

Classification	Definition
High-risk PTE	Persistent hypotension, defined as systolic blood pressure < the lower limit in the same age group (within 1 mon: < 60 mmHg; from 1 mon to 1 y old: < 70 mmHg; from 1 y old to 10 y old: < 70 + (age × 2) mmHg; over 10 y old: < 90 mmHg) or need for vasoactive drug support or pulselessness or sustained heart rate < 60 beats/min with signs/symptoms of shock or compensated (normotensive) shock
Intermediate-risk PTE	Systolic blood pressure > the low limit in the same age group, but right ventricular dysfunction or myocardial necrosis, manifested by any of the following: right ventricular dilatation (echocardiogram or CT); echocardiography shows right ventricular systolic dysfunction; elevated troponin
Low-risk PTE	Acute PTE and lack of clinical signs, symptoms and biomarkers defined as high- or intermediate-risk PTE

*PTE* pulmonary thromboembolism, *CT* computer tomography

The original source of the classification can be found in Refs [29, 30]

### Pathophysiology

#### Thrombosis

Factors such as stagnant venous blood flow, vascular endothelial damage, and blood hypercoagulability contribute to the formation of thrombosis. Thrombi originating from the peripheral venous system may detach and migrate to the pulmonary artery, leading to PTE. In addition, focal inflammation and endothelial dysfunction of the pulmonary vasculature caused by pulmonary infectious diseases, pulmonary trauma, or pulmonary vasculitis can result in in-situ pulmonary thrombosis [31]. In recent years, the incidence of severe *Mycoplasma pneumoniae* (MP) pneumonia in children has increased. Moreover, the combination of MP pneumonia with PTE is more frequently reported [15, 32]. Thrombosis in these cases may be associated with direct MP invasion, indirect immune-mediated injury, vasculitis, and hypercoagulability [31].

#### Increased pulmonary vascular resistance and cardiac insufficiency

At the onset of PTE, thromboembolic obstruction of the pulmonary arterial bed can lead to increased pulmonary arterial pressure. Anatomical obstruction with hypoxic vasoconstriction results in elevated pulmonary vascular resistance and reduced arterial compliance [1, 4, 12]. Increased pulmonary vascular resistance causes right ventricle (RV) dilatation, which impairs myocardial contractile properties. Elevated atrial pressure and volume increase ventricular wall tension and stretch cardiomyocytes. Prolonged atrial contraction, neurohumoral activation, and systemic vasoconstriction temporarily elevate pulmonary arterial pressure to stabilize systemic blood pressure. However, when RV diastolic pressure exceeds that of the left ventricle (LV), the interventricular septum deviates leftward, impairing LV filling. Meanwhile, reduced RV output results in diminished pulmonary venous return to the LV, further decreasing LV preload. These changes lead to compromised LV output, systemic hypotension, and shock [33].

#### Respiratory insufficiency

In the presence of hemodynamic instability, cardiac output decreases, leading to a reduction in mixed venous oxygen saturation. Pulmonary blood flow decreases at embolized sites while perfusion increases at unembolized sites, resulting in a ventilation–perfusion mismatch and ultimately hypoxemia. In about one-third of children, elevated right atrial pressure can cause reopening of the foramen ovale with subsequent right-to-left shunting, leading to severe hypoxemia. If the embolus occludes a small distal vessel, it may result in local hemorrhagic pulmonary atelectasis and alveolar hemorrhage, further impairing gas exchange [1, 4].

#### Chronic thromboembolic pulmonary hypertension

Following thromboembolic obstruction of the pulmonary artery, thrombus insolubility, organization, and pulmonary vascular remodeling can lead to vessel stenosis or occlusion. This results in increased pulmonary vascular resistance and a progressive rise in pulmonary arterial pressure, ultimately causing right ventricular hypertrophy and right heart failure, a condition known as chronic thromboembolic pulmonary hypertension (CTEPH).

## Diagnosis of pulmonary thromboembolism

### Question 1: What clinical manifestations should be suspected of pulmonary thromboembolism in children?

#### Recommendation

Symptoms in children: dyspnea or shortness of breath is the most common symptom. While dyspnea, chest pain, and hemoptysis are the typical triad of pulmonary embolism, fewer than 30% of children present with this typical triad simultaneously. In addition, common symptoms in children include cough, syncope, tachycardia, and hypoxia. Other symptoms include fever, palpitations, other types of arrhythmias, and hypotension (very low quality, weak recommendation).

Physical signs in children: consider PTE when the following physical signs suddenly appear without apparent causes: (1) shortness of breath; (2) tachycardia; (3) changes in blood pressure or progression to shock; (4) cyanosis; (5) fever, mostly low; (6) jugular vein distension or pulsation; (7) wheezing and (or) moist rales on lung auscultation, occasionally with vascular murmurs; (8) corresponding signs of pleural effusions; (9) hyperactivity or splitting of the second heart sound at the pulmonary valve (P2 > A2), tricuspid systolic murmur (good practice statement).

#### Summary of the evidence

Clinical symptoms and physical signs of children with PTE are diverse and non-specific. Our meta-analysis showed that common clinical manifestations in children with PTE (including nonverbal children) are dyspnea, cough, chest pain, shortness of breath, and hemoptysis [15, 25, 34–42] (Supplementary Table S1). Nevertheless, the relatively small sample sizes for the typical triad, shortness of breath, and hemoptysis should be acknowledged, as this limitation may reduce the robustness of these findings.

### Question 2: How can pulmonary thromboembolism be diagnosed initially in children?

#### Recommendation

Assess children with suspected PTE based on experience and clinical likelihood rating scales when available. For children with a high probability on clinical evaluation, a definitive diagnostic test may be considered. For those with a low clinical probability, D-dimer levels could be helpful. If the D-dimer level is below the thrombotic threshold, usually < 0.5 mg/L (fibrinogen equivalent units), acute PTE is less likely. If the D-dimer level is elevated, a definitive diagnostic test might be warranted (very low quality, weak recommendation).

#### Summary of the evidence

Studies on children with PTE demonstrated the poor sensitivity and specificity of existing clinical likelihood rating scales (i.e., pulmonary thrombosis exclusion criteria and pediatric thrombosis decision rules) for the diagnosis of PTE [25, 42, 43]. Evidence showed [44, 45] that the use of a clinical likelihood rating score combined with D-dimer testing reduced the rate of missed PTE diagnosis (Supplementary Table S2). It should also be acknowledged that current evidence supporting the use of clinical likelihood rating scores is derived from studies of PE in adults, and their diagnostic utility in children with PTE may therefore be limited. However, due to the potential for misdiagnosis in D-dimer testing [46–51] (Supplementary Table 3), the expert panel suggested caution when interpreting D-dimer results.

### Question 3: How can pulmonary thromboembolism be definitively diagnosed in children?

#### Recommendation

Where condition permits, perform computed tomography pulmonary angiography (CTPA) to confirm or exclude the diagnosis of PTE (very low quality, weak recommendation).

For children with suspected PTE and hemodynamic instability, if CTPA is unavailable or inappropriate: (1) perform bedside echocardiography. If increased right ventricular load and/or thrombus in the pulmonary artery or right ventricle is detected, diagnose PTE after ruling out other potential diseases. (2) Perform limb compression venous ultrasound. Confirm PTE if evidence of DVT is found. Once the clinical condition stabilizes, perform relevant tests to clarify the consideration (very low quality, weak recommendation).

For children with suspected PTE and stable hemodynamics where CTPA is contraindicated (i.e., iodine contrast agent allergy and renal insufficiency), consider alternative imaging options, including pulmonary ventilation/perfusion (V/Q) imaging and magnetic resonance pulmonary arteriography (MRPA) (very low quality, weak recommendation).

#### Summary of the evidence

The plain chest radiograph, a commonly used first-line examination, offers limited value in diagnosing PTE. About 80% of children with PTE present with abnormal chest radiographic findings, while 12%–24% may have normal results. The most common abnormalities include right heart enlargement (27%), pleural effusion (23%), pulmonary atelectasis (18%), and pulmonary consolidation (17%). Given that these findings are non-specific, PTE should be considered after ruling out other potential diagnoses.

CTPA has become the preferred method for pulmonary angiography in children with suspected PTE due to its high diagnostic accuracy. However, the diagnostic threshold setting for CTPA can affect its sensitivity and specificity in children with PTE [52]. Based on current evidence, the expert panel recommended less than 22 Hounsfield Units (HU) as the diagnostic threshold (Supplementary Table 4). Clinicians should be cautious when clinical probability is inconsistent with CTPA findings. In children with low clinical suspicion of PTE, a negative CTPA can rule out PTE, whereas a positive result warrants further investigation (especially if the thrombus is confined to a segmental or subsegmental vessel). In children with high or intermediate clinical suspicion of PTE, CTPA can confirm the diagnosis if it shows partial or complete low-density filling defects in pulmonary vessels at or above the segment level, or complete filling defects with non-visible distal vasculature. Indirect signs such as high-density wedge-shaped bands or discoid atelectasis in the pulmonary field, central pulmonary artery dilatation, and reduced or absent distal vascular distribution may also suggest PTE. For cases involving single or distal subsegmental PTE, further confirmation should involve lower extremity venous ultrasound, V/Q scanning, or pulmonary angiography.

No evidence supported the use of MRPA in children with PTE. However, studies conducted in adult patients with PTE demonstrated high sensitivity and specificity for MRPA [53] (Supplementary Table 5). Various MRPA techniques can be used to evaluate the pulmonary vasculature. Time-resolved enhancement techniques offer a speed advantage, making this particularly suitable for imaging children.

Similarly, no relevant evidence supported the use of ventilation–perfusion (V/Q) imaging in children with PTE. However, the expert panel agreed that V/Q imaging was an established and reliable diagnostic method with significant advantages. It is safe, rarely triggers allergic reactions, and involves much lower radiation exposure compared to CTPA. The panel recommended V/Q imaging for children older than six years who could cooperate with respiratory maneuvers. Diagnostic criteria for PTE on V/Q imaging include a mismatch between perfusion and ventilation images, where tracer distribution in the perfusion image is sparse or defective, while the ventilation image shows no abnormalities.

## Treatment of pulmonary thromboembolism

### Question 4: Should anticoagulation be administered for children with pulmonary thromboembolism?

#### Recommendation

Administer anticoagulant therapy to children with symptomatic PTE (very low quality, weak recommendation). For children with asymptomatic PTE, consider anticoagulant therapy based on individual clinical condition (good practice statement).

In cases where children with PTE are not initially receiving anticoagulant therapy and radiologic monitoring reveals progression of thrombosis without adequate control of risk factors, initiate anticoagulation alongside relevant laboratory test results (good practice statement).

#### Summary of the evidence

In children with symptomatic DVT or PTE, the evidence was primarily from single-arm studies, reporting 3.6% (2/55, number of events/number of patients) of mortality [18] and 0.9% of major bleeding [95% confidence interval (CI) 0.0%–5.9%] [54–59] in the anticoagulant therapy group (Supplementary Table 6). Studies in children with symptomatic or asymptomatic PTE are also single-arm and reported 0.3% of PTE exacerbation (95% CI 0.0%–2.1%) [54, 55, 60] and 1.7% of VTE recurrence (95% CI 1.0%–2.5%) in the anticoagulant therapy group. Only two studies compared anticoagulation with no anticoagulation. A cross-sectional study found anticoagulation is associated with a higher risk of major bleeding [risk ratio (RR) 4.09, 95% CI 0.19–86.09] [56–59]; however, the power to detect differences between groups was low due to the small sample size. An randomized clinical trial (RCT) in adults with PTE found that the mortality or recurrence of VTE is lower in adults who were anticoagulated than in those who were not [1.85% (1/54) vs. 26.3% (5/19), RR 0.07, 95% CI 0.01–0.57] [51] (Supplementary Table 6).

For children with asymptomatic PTE, the evidence was primarily from single-arm studies, making it difficult to compare the benefits and harms between anticoagulant therapy and no treatment (Supplementary Table 7). As a result, the expert panel considered that the decision to use anticoagulant therapy should depend on clinical condition.

In cases of children with PTE who were not receiving anticoagulant therapy, and in whom radiologic monitoring showed thrombosis extension, no evidence showed whether anticoagulation should be initiated. Considering that anticoagulants provided preventive and therapeutic benefits, and most children with PTE and indications for anticoagulation would consider the potential benefits to outweigh the potential harms, the expert panel recommended starting anticoagulant therapy based on laboratory test results.

### Question 5: How to choose anticoagulant drugs for children with pulmonary thromboembolism?

#### Recommendation

In children with symptomatic PTE, consider low molecular weight heparin (LMWH), unfractionated heparin (UFH), or Vitamin K antagonists (VKA) (very low quality, weak recommendation).

In children with PTE who do not respond to these treatments, antithrombin replacement therapy may be considered; however, antithrombin replacement therapy should not be considered as the first-line treatment option (very low quality, weak recommendation).

#### Summary of the evidence

In children with DVT or PTE, an RCT indicated that LMWH might increase the risk of minor bleeding (defined in the context of the primary underlying disorder and what was “usually” expected, such as, but not exclusive to, bruising or oozing around intravenous sites and surgical wounds, small amounts of blood from suctioning endotracheal tubes, small amounts of blood in urine or stool, and minor nose bleeds) and any other bleeding compared to other anticoagulants, such as UFH or warfarin [61]. However, studies found no significant differences in mortality, incidence of PTE or DVT, overall bleeding rates [62] or major bleeding rates [61] (Supplementary Table 8). However, for the evidence for children with PTE, the power to detect differences between groups was low due to the small sample size. Studies in adults with DVT or PTE, which can provide indirect evidence, similarly found no significant differences between UFH and LMWH in terms of PTE, VTE recurrence, bleeding, or mortality, while LMWH was less costly [56, 63–65] (Supplementary Table 8).

In children with DVT or PTE, there were no significant differences in mortality, PTE exacerbation, or the incidence of major bleeding between LMWH and VKA [61] (Supplementary Table 9), but the power to detect differences between groups was low due to the small sample size. The expert panel agreed that anticoagulant selection should be based on the severity and remission of PTE. Parenteral administration is preferred for acute resuscitation; newer oral anticoagulants such as rivaroxaban and dabigatran may be considered for remission and maintenance bridging [66–68].

In children with VTE, we only found one study that compared children with PTE treated with combined antithrombin therapy or standard anticoagulant therapy. This study has not been published and showed that combined antithrombin therapy increased the time to therapeutic anti-Xa, increased cost and increased bleeding (14.3% vs. 3.9%; *P* = 0.55) [60]. We included additional studies in our meta-analysis to provide indirect information for the use of combined antithrombin therapy [69–76] (Supplementary Table 10); however, the power was limited due to the indirectness. In terms of major bleeding, our meta-analysis of indirect evidence from RCTs in children found no significant difference between combined antithrombin therapy and standard anticoagulant therapy. However, our meta-analysis of cohort children studies showed that antithrombin therapy was associated with an increased rate of major bleeding [70, 74–77] (Supplementary Table 10).

### Question 6: How to determine the course of anticoagulant therapy for children with pulmonary thromboembolism?

#### Recommendation

Continue anticoagulant therapy for 3–12 months in children with PTE. Adjust duration based on etiology, clinical presentation, and imaging findings (good practice statement). Children with idiopathic VTE should receive anticoagulant therapy for 6–12 months (very low quality, weak recommendation). For children with recurrent idiopathic VTE, extend the duration of anticoagulant therapy (good practice statement). In children with secondary VTE, anticoagulant therapy should continue for at least three months if the risk factors have been resolved. If reversible risk factors persist, therapy should extend beyond three months until those risk factors are eliminated (very low quality, weak recommendation). For children with recurrent secondary VTE, continue anticoagulant therapy for more than three months if reversible risk factors remain, until those factors are resolved (good practice statement).

Monitor medication and coagulation parameters regularly throughout the course of therapy for children with PTE (good practice statement).

#### Summary of the evidence

In children with idiopathic DVT or PTE, studies did not demonstrate significant differences in VTE recurrence rates with varying durations of anticoagulant therapy [78] (Supplementary Table 11), but the power to detect differences between groups was low due to the small sample size.

For children with recurrent idiopathic PTE, clinical evidence remains limited. The expert panel emphasized the importance of assessing the risks of recurrence and bleeding and suggested consideration of long-term anticoagulant therapy with regular follow-up when indicated.

In children with secondary DVT or PTE, a single-center cohort study failed to detect differences in recurrence rates across different durations of anticoagulant therapy [78] (Supplementary Table 12). For children treated with LMWH or VKA for 3–6 months, a six-month follow-up RCT reported a VTE recurrence rate of 7.9% (6/76), a major bleeding incidence of 9.2% (7/76), and a mortality rate of 6.6% (5/76) [79] (Supplementary Table 12), but the power to detect differences between groups was low due to the small sample size. Based on these findings, the expert panel concluded that anticoagulant therapy should be individualized for children according to treatment outcomes and risk factor assessments.

For children with recurrent secondary PTE, the evidence was insufficient. The expert panel agreed to focus on identifying the causes of recurrence, assessing the risk of recurrence and bleeding, and continuing anticoagulant therapy until the primary disease was resolved or stabilized.

Furthermore, the expert panel concurred that for children with PTE, medication monitoring should occur throughout anticoagulant therapy. For children with PTE receiving warfarin, limited clinical studies, alongside in vitro studies, suggested that the intensity of anticoagulation should be lower than in adults. Based on adult data, pediatric patients on warfarin therapy should maintain an international normalized ratio (INR) of 2.5 [80, 81]. For children with PTE treated with UFH, adjust the dosage to achieve an anti-Xa activity level between 0.35 and 0.70 IU/mL, or an activated partial thromboplastin time (aPTT) range that correlates with this anti-Xa range, or alternatively, a protamine titration range of 0.2–0.4 U/mL [82]. For neonates and children receiving once- or twice-daily LMWH, target anti-Xa activity should range from 0.5 to 1.0 U/mL for samples taken 4–6 hours after subcutaneous injection or 0.5 to 0.8 U/mL in a sample taken 2–6 hours after subcutaneous injection [81]. For children with PTE receiving rivaroxaban, adjust the dosage according to age and weight to match an equivalent adult dose of 20 mg. For children with PTE under six months who take rivaroxaban three times daily, after 30 consecutive days of treatment, the prothrombin time should be 1.35 [standard deviation (SD) 0.20] times the baseline; the aPTT should be 1.31 (SD 0.15) times the baseline and the anti-Xa activity should measure 118.12 (SD 82.08) μg/L [68].

### Question 7: Should thrombolytic therapy be administered for children with pulmonary thromboembolism?

#### Recommendation

Consider thrombolytic therapy for children with high-risk PTE (i.e., hemodynamically unstable) who have no contraindications to thrombolysis (very low quality, weak recommendation).

Thrombolysis in intermediate-risk PTE in children could not be assessed due to a lack of evidence.

#### Summary of the evidence

For children with high-risk PTE, a systematic review found no significant differences between the active treatment and observation groups in the incidence of all-cause hospital mortality, PE-related mortality, fatal major bleeding, or nonfatal major bleeding [23, 35, 82]; however, the power to detect differences between groups was low due to the small sample size (Supplementary Table 13). For children with intermediate-risk PTE, a systematic review found no significant differences between the thrombolytic therapy group and the anticoagulant only or none group for nonfatal major bleeding. The incidence of all-cause hospital mortality (0/13 vs. 4/20), PE-related mortality (0/13 vs. 2/20), fatal major bleeding (0/13 vs. 1/2), and CTEPH (0/13 vs. 2/20) may be lower in thrombolytic therapy, but the evidence is insufficient for the small number of children [23, 35, 82]; however, the power to detect differences between groups was low due to the small sample size (Supplementary Table 13). In one study which did not differentiate PTE risk categories, combined anticoagulation and thrombolytic therapy was associated with a higher rate of major bleeding when compared to the anticoagulation only or none group [18]; however, indication bias is likely significant in this cohort, and the power to detect differences between groups was low due to the small sample size.

For children with VTE (without risk level stratification), most of the included studies only focused on combined thrombolytic therapy. Only one cohort study found that thrombolytic therapy may be associated with a higher incidence of major bleeding (1/9 vs. 0/13) and a lower incidence of PTS (1/9 vs. 8/13) compared with the anticoagulant only or none group; however, the power to detect differences between groups was low due to the small sample size [83].

Moreover, our meta-analysis showed that the influence on all-cause mortality, DVT, post-thrombotic syndrome (PTS), and the incidence of major bleeding is unclear [84–95] (Supplementary Table 13). Two cross-sectional studies reported outcomes in 14 children with PTE who underwent thrombolytic therapy, of whom three required additional interventions; seven experienced major bleeding; three died, and one developed pulmonary hypertension [18, 96] (Supplementary Table 13).

In the survey of preference of the expert panel, the expert panel concluded that most children with PTE and indications for thrombolytic therapy’s potential benefits should outweigh the potential harms, and most children would prefer to receive thrombolytic therapy.

### Question 8: How to select thrombolytic drugs for children with pulmonary thromboembolism with indications for thrombolysis?

#### Recommendation

For children with PTE, use tissue-type plasminogen activator (tPA) as the preferred agent for thrombolytic therapy. Consider urokinase and streptokinase as potential alternatives (good practice statement).

#### Summary of the evidence

Clinical research on the selection of thrombolytic agents for children with PTE remained limited. However, based on expert consensus, the panel agreed that tPA was the preferred thrombolytic agent due to its favorable balance of efficacy and safety. Urokinase and streptokinase were potential alternatives. The most commonly reported dosing regimen for tPA was 0.1–0.6 mg/kg/hour, with dosage reassessment typically conducted after six hours [80].

### Question 9: Should catheter-based interventional therapy be considered for children with pulmonary thromboembolism?

#### Recommendation

Consider percutaneous catheter-based interventional therapy (i.e., catheter-directed thrombolysis or catheter-based thrombus aspiration) as an alternative to thrombolysis for high-risk children with thrombus present in the pulmonary artery trunk or major branches and for those with a high risk of bleeding, contraindications to thrombolysis, or failure of thrombolysis or aggressive medical therapy (very low quality, weak recommendation).

For children with low-risk PTE, catheter-based interventions are generally not recommended. Instead, consider catheter-directed thrombolysis or catheter embolectomy based on factors such as vital signs, gas exchange, and the presence of contraindications (good practice statement).

Catheter-based interventional therapy in intermediate-risk PTE in children could not be assessed due to a lack of evidence.

#### Summary of the evidence

In children with PTE, a case series showed that catheter-directed thrombolysis could alleviate clinical symptoms with no reports of mortality, bleeding, or CTEPH. In children with high-risk PTE, our meta-analysis of two cohort studies showed no differences in all-cause hospital mortality and PE-related mortality between catheter-directed thrombolysis and systemic thrombolysis, and no fatal major bleeding, nonfatal major bleeding, or CTEPH occurred in catheter-directed thrombolysis; however, the power to detect differences between groups was low due to the small sample size (Supplementary Table 14). In children with intermediate-risk PTE, our meta-analysis found that no death, fatal major bleeding, nonfatal major bleeding, or CTEPH occurred in catheter-directed thrombolysis; however, the power to detect differences between groups was low due to the small sample size (Supplementary Table 14). In adults with PTE, several studies have also demonstrated that catheter-directed thrombolysis could effectively relieve clinical symptoms with a relatively low incidence of adverse events such as bleeding and death [18, 85, 88, 89, 97–100]; however, indication bias is likely significant in these studies (Supplementary Table 15). Due to a lack of clinical evidence on catheter embolectomy in children, the expert panel based its recommendations on clinical experience and expert consensus.

### Question 10: Should surgical embolectomy be performed for children with pulmonary thromboembolism?

#### Recommendation

Consider surgical embolectomy for children with high-risk PTE under the following circumstances: (1) Treatment: failed or contraindicated anticoagulant or thrombolytic therapy. (2) Age: neonates or premature infants, in whom anticoagulant and thrombolytic therapy may carry an elevated risk of bleeding. (3) Specific pulmonary anatomy: PTE occurring after palliative surgeries (such as the Blalock–Taussig shunt or Fontan procedure) in children with single ventricles. (4) Embolus characteristics: tumorous embolism or embolism accompanied by right ventricular thrombus (very low quality, weak recommendation).

Surgical embolectomy in intermediate-risk PTE in children could not be assessed due to a lack of evidence.

#### Summary of the evidence

Our pooled analysis included four case studies that involved eight children; seven underwent surgical embolectomy, of which four had PTE after palliative surgery (Fontan procedure) [101]; one with tumorous embolism [102], one with failure of anticoagulant or thrombolytic therapy [103] and one premature infant with a high risk of intracranial hemorrhage [104] (Supplementary Table 16).

A case–control study included five children, and one underwent surgical embolectomy due to failure of anticoagulant or thrombolytic therapy [105] (Supplementary Table 16).

Our pooled analysis included two cohort studies that included 87 children; seven underwent surgical embolectomy, of which five had tumorous embolism [23] and two had intracranial hemorrhage [18] (Supplementary Table 16).

However, the power of evidence was low due to the small sample size of the above studies.

In the survey of preference of expert panel, the panel agreed that most children with PTE with indications for surgical embolectomy would consider the potential benefits to outweigh the potential harms and that most children would prefer to receive surgical embolectomy.

### Question 11: What other supportive treatments should be considered for children with pulmonary thromboembolism?

#### Recommendation

Provide appropriate respiratory and circulatory management for children with PTE (very low quality, weak recommendation).

#### Summary of the evidence

Limited evidence exists on respiratory and circulatory management in children with PTE. According to relevant guidelines for adults with PTE [106], respiratory management should include oxygen therapy and low tidal volume ventilation. For circulatory management, the primary focus should center on supporting blood pressure and adequate perfusion. Consider vasoactive agents as needed.

### Question 12: Should a pulmonary embolism response team be established for children with acute pulmonary thromboembolism?

#### Recommendation

Consider the establishment of a pulmonary embolism response team (PERT) to manage acute PTE (very low quality, weak recommendation).

#### Summary of the evidence

A PERT typically comprises a multidisciplinary team involving various clinical specialties, such as emergency medicine, cardiology, respiratory medicine, cardiovascular surgery, critical care medicine, pharmacy, and radiology [107, 108]. The team is responsible for the initial assessment, diagnosis, treatment, outpatient care, and follow-up of patients with PTE [109].

One cohort study compared the health outcomes of children with PTE before and after PERT [108]. This study has shown that a PERT may increase the proportion of children who are eligible and receive reperfusion, shorten the time to echocardiogram, time to anticoagulation, and time to anticoagulation order. Furthermore, there were consistently no significant differences in major bleeding rates or PE-related mortality. However, the power to detect differences between groups was low due to the small sample size.

In adults with PTE, cohort studies showed that the establishment of a PERT could increase the proportion of patients receiving advanced treatment, such as catheter-directed therapies [109] and improve reperfusion rates. In addition, PERT could shorten intensive care unit stay [110], triage-to-diagnosis time, diagnosis-to-treatment time, and diagnosis-to-reperfusion time [111]. Despite these benefits, no significant differences were consistently observed in major bleeding rates, overall mortality [109] or 30-day rehospitalization risk [112]. Findings on the impact of PERT on major bleeding rates, mortality, or in-hospital mortality were inconsistent among studies (Supplementary Table 17).

## Treatment of pulmonary thromboembolism with comorbidities

### Question 13: How to treat pulmonary thromboembolism combined with *Mycoplasma pneumoniae* pneumonia in children?

#### Recommendation

Children with MP pneumonia presenting with positive antiphospholipid antibodies, abnormal anticoagulant proteins, elevated D-dimer, lobular consolidation, and pulmonary necrosis should be highly vigilant against PTE (very low quality, weak recommendation).

Children with MP pneumonia complicated with PTE should receive anticoagulant therapy. Consider thrombolytic therapy for high-risk PTE cases (very low quality, weak recommendation).

#### Summary of the evidence

PTE is more frequently associated with MP pneumonia in children than in adults. A case series of children with MP pneumonia combined with PTE showed that the onset of PTE did not necessarily align with the acute phase of MP infection. Moreover, symptoms such as dyspnea and chest pain during disease remission warranted careful evaluation [32]. The clinical presentation of MP pneumonia combined with PTE often mimics primary pulmonary conditions leading to potential under-recognition or misdiagnosis [37].

Refractory MP pneumonia associated with positive antiphospholipid antibodies, abnormal anticoagulant protein levels, elevated D-dimer, and radiologic findings of lobular consolidation and pulmonary necrosis should prompt suspicion of thrombosis [37, 40, 113, 114]. Emboli can occur in any segment of the pulmonary artery but are most commonly found in the right pulmonary lobe or segmental arteries [113].

Case series for children with MP pneumonia complicated with PTE showed good effectiveness and safety for anticoagulation [37, 114–117]. Thrombolytic therapy might benefit some critically ill cases, but further multicenter, large-sample studies are needed for indications, administration routes, dosages, and duration of thrombolytic therapy for children [114] (Supplementary Table 18). However, the small sample size may have introduced variability into the results.

### Question 14: How to treat pulmonary thromboembolism during the perioperative period in children?

#### Recommendation

For children with acute high-risk PTE during the perioperative period: (1) If PTE occurs within one week of surgery, thrombolytic therapy should generally be avoided due to the risk of bleeding. However, in life-threatening situations, such as cardiac arrest, thrombolytic therapy (i.e., tissue plasminogen activator, tPA) or other treatments (i.e., interventional or surgical thrombectomy) may be considered. (2) If PTE occurs more than one week after surgery, thrombolytic therapy may be considered depending on clinical status and bleeding risk.

For children with PTE on anticoagulant therapy who require surgery: (1) For pediatric patients receiving warfarin with a high risk of PTE recurrence and no significant bleeding tendency, discontinuation of warfarin and initiation of bridging anticoagulation about five days before surgery should be individualized. This should be based on factors such as recurrence history, underlying thrombophilia, recent thrombotic events, or the presence of malignancy. (2) For children receiving parenteral anticoagulation (i.e., UFH or LMWH) or bridging anticoagulation, discontinuation 4–6 hours before surgery may be appropriate. Continuing anticoagulant therapy may be considered for low-bleeding-risk procedures, such as minor oral, dermatologic, or cataract surgery (good practice statement).

#### Summary of the evidence

Evidence on the management of children with PTE during the perioperative period is limited, and the expert panel developed its recommendations based on clinical experience. Importantly, bridging anticoagulation is an important strategy during the perioperative period, using short-acting anticoagulants (UFH or LMWH) as a substitute for long-acting drugs (VKA). For children receiving long-term oral anticoagulants (i.e., warfarin) or antiplatelet therapy who require surgical or invasive procedures, discontinuation of antithrombotic medications preoperatively increases the risk of thromboembolism. Conversely, continuing such therapy during the perioperative period significantly increases the risk of postoperative bleeding. Bridging anticoagulation offers a balanced approach to mitigate both risks.

### Question 15: How to treat pulmonary thromboembolism combined with renal impairment or renal failure in children?

#### Recommendation

For children with confirmed PTE and renal impairment (creatinine clearance 15–50 mL/minute): (1) apixaban, (2) rivaroxaban, and (3) LMWH for at least five days followed by edoxaban or dabigatran, provided creatinine clearance exceeds 30 mL/minute, are recommended. For children with confirmed PTE and renal failure (creatinine clearance < 15 mL/minute): (1) LMWH, (2) UFH, and (3) LMWH or UFH in combination with VKA for at least five days initially, or until the INR reaches at least 2.0 for two consecutive readings, followed by VKA alone, are recommended (good practice statement).

#### Summary of the evidence

For children with renal impairment or renal failure combined with PTE, studies showed that treatment with standard anticoagulant bridging warfarin with UFH or LMWH was effective in relieving symptoms [118, 119] (Supplementary Table 20). However, the small sample size may have introduced variability into the results.

## Prevention of pulmonary thromboembolism recurrence

### Question 16: Should a venous filter be placed for children with pulmonary thromboembolism?

#### Recommendation

A venous filter should be avoided unless absolutely necessary. For children with high-risk PTE, contraindications to systemic anticoagulation, VTE in the lower extremities, and body weight ≥ 10 kg, placing a retrievable venous filter can be considered (very low quality, weak recommendation).

#### Summary of the evidence

In children with DVT or PTE, our meta-analysis showed a mortality rate of 8% (95% CI 0.0%–4.7%) in the inferior vena cava (IVC) filter group [120–127], and a previous guideline reported mortality ranging from 0% to 6% for children and adults with other conditions [128]. Besides, our meta-analysis did not find that major bleeding occurred in the IVC group [120, 121, 126, 127]. However, IVC filter placement might lead to adverse events such as venous thrombosis or failure to retrieve the filter due to adherent thrombus [18, 120–127] (Supplementary Table 19).

The expert panel agreed that, given the clinical benefits and technical challenges associated with venous filter placement, particularly in children, their use should be avoided unless absolutely necessary. Indications for venous filter placement include high-risk PTE, contraindications to systemic anticoagulation (i.e., bleeding tendencies or platelet dysfunction), and the presence of VTE in the lower extremities. In addition, consider individual patient and family preferences.

### Question 17: When should venous filters be removed for children with pulmonary thromboembolism placed with venous filters?

#### Recommendation

Remove venous filters in children with PTE as soon as contraindications to anticoagulation are resolved and promptly resume anticoagulant therapy (good practice statement).

#### Summary of the evidence

No evidence specified the optimal timing for venous filter removal. The expert panel developed the recommendation based on experience and expert consensus.

### Question 18: How to evaluate the prognosis for children with pulmonary thromboembolism?

#### Recommendation

Initial risk stratification in children with suspected or confirmed acute PTE should prioritize identifying high-risk patients at risk of early death by assessing for shock or persistent hypotension. For non-high-risk children without shock or persistent hypotension, a clinical prognostic risk score can differentiate between intermediate- and low-risk cases (good practice statement).

#### Summary of the evidence

Evidence on prognostic assessment in children with PTE was limited. Based on clinical experience, the expert panel agreed to utilize a prognostic risk score, such as the Pulmonary Embolism Severity Index (PESI) [4], supplemented by clinical, imaging, and laboratory parameters related to PTE prognosis.

### Question 19: How to conduct health education for children with pulmonary thromboembolism?

#### Recommendation

Encourage early mobilization in hemodynamically stable children with acute PTE, once adequate anticoagulation is achieved. Provide comprehensive medication and discharge education to children and their families to enhance medication adherence (good practice statement).

#### Summary of the evidence

No evidence addressed health education for children with PTE. The recommendation, based on clinical experience, emphasized the importance of early mobilization and education to optimize treatment outcomes and improve adherence.

### Question 20: How long should the follow-up period be after anticoagulant therapy for children with pulmonary thromboembolism?

#### Recommendation

Monitor children with PTE who are eligible for long-term follow-up continuously after completing anticoagulant therapy. For those not eligible for long-term follow-up, monitor for at least three months after anticoagulant therapy. If relapse occurs, extend follow-up for at least three months after the recurrence (good practice statement).

#### Summary of the evidence

Evidence on the follow-up period for children with PTE was limited, and the expert panel developed the recommendation based on experience.

## Conclusions

**Fig. 1:**
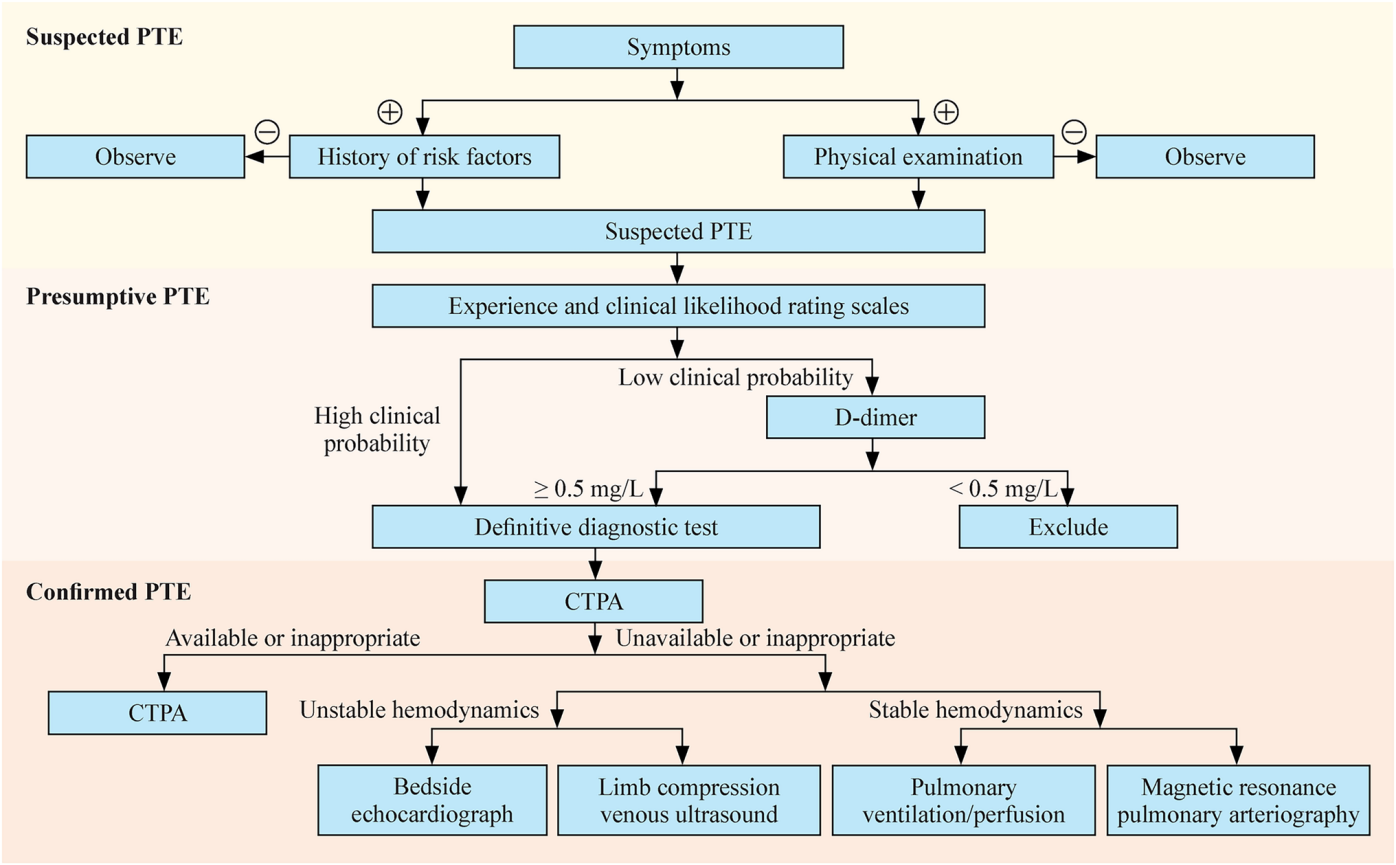
Flow chart of the process of diagnosis recommended in this evidence-based expert consensus. *PTE* pulmonary thromboembolism, *CTPA* computed tomography pulmonary angiography

**Fig. 2:**
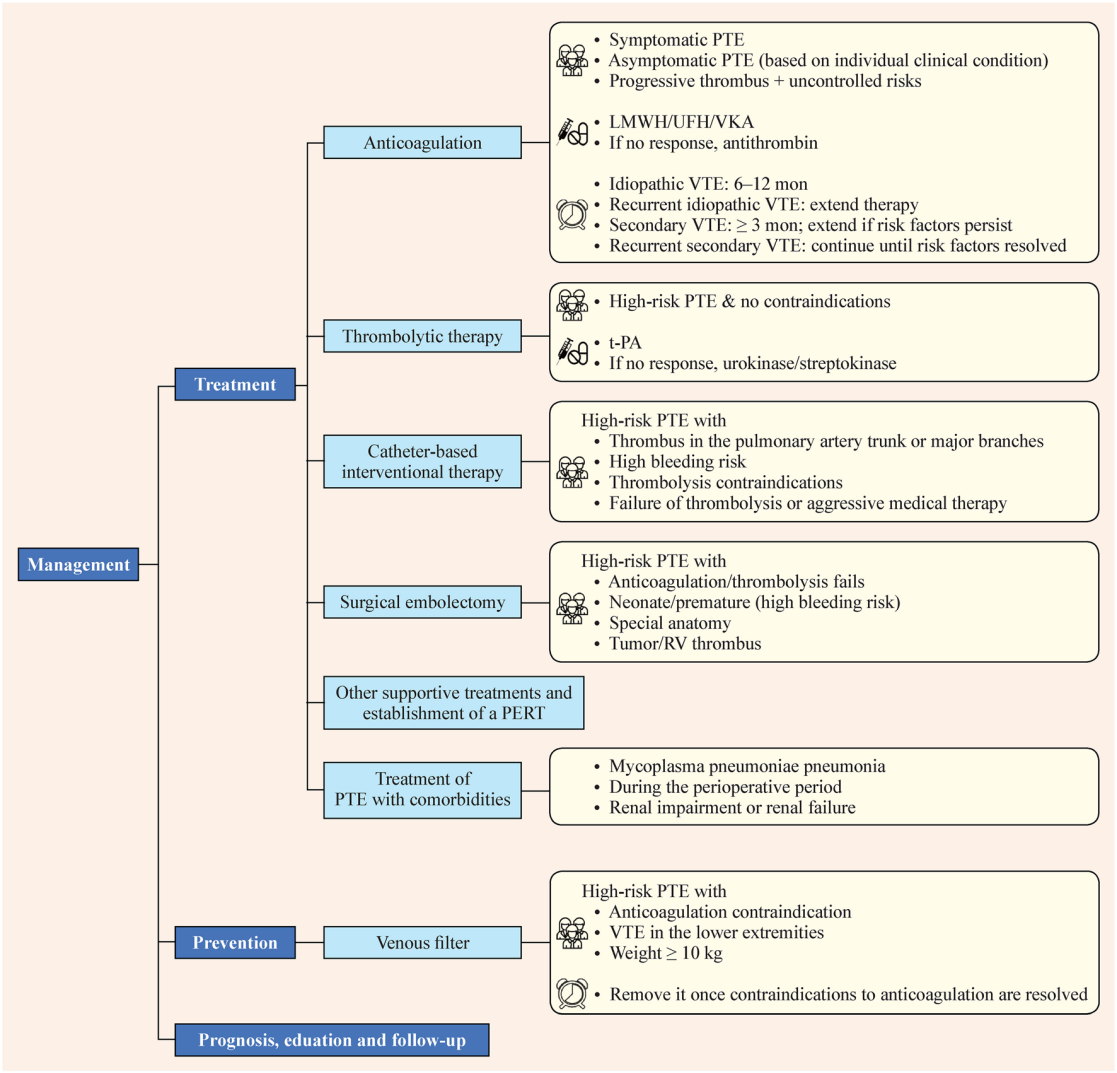
Flow chart of the process of management of PTE recommended in this evidence-based expert consensus. *PTE* pulmonary thromboembolism, *LMWH* low molecular weight heparin, *UFH* unfractionated heparin, *VKA* Vitamin K antagonists, *VTE* venous thromboembolism, *tPA* tissue-type plasminogen activator, *RV* right ventricle, *PERT* pulmonary embolism response team

This expert consensus based on available evidence provides a detailed and comprehensive set of recommendations for clinicians involved in the diagnosis (Fig. 1) and management (Fig. 2) of PTE in children. It covers key aspects of PTE in children, including overviews, diagnostic strategies, treatments, and more.

During formulation, the expert panel conducted a systematic search on the epidemiology, diagnosis, treatment, and nursing of PTE in children. We found that clinical research evidence was insufficient, and the quality of that evidence was generally low. Most recommendations were based on reasonable extrapolation of data from clinical research on PTE in adults and the clinical experience of experts, considering the values and preferences of the children and their parents. The expert panel reached a consensus and formed a Statement of Good Practices.

The expert panel calls on clinicians and researchers to focus on PTE in children and to study early identification and diagnostic strategies, prevention and therapeutic regimens, and long-term management. Through in-depth exploration and rigorous research to improve the understanding of PTE in children, the expert panel hopes that we can provide more accurate and efficient clinical management strategies for children with PTE in the future.

## Supplementary Information

Below is the link to the electronic supplementary material.

Supplementary file1 (DOCX 424 KB)

## Data Availability

The data are available as electronic supplementary material.
