# Supplementary material for: Management of pulmonary thromboembolism in children: an evidence-based expert consensus
Source: World J Pediatr. 2026 Feb 20;22(3):330–48. doi: 10.1007/s12519-025-00987-3 (PMC13076377; doi:10.1007/s12519-025-00987-3)
Supplement: Supplementary file 1 — Supplementary file1 (DOCX 424 KB) [file 12519_2025_987_MOESM1_ESM.docx]

**Appendix 1. The list of the expert panel and secretary group (in alphabetical order by last name)**

**1 The guideline panel**

**The steering group:**

Quan Lu (Shanghai Children's Hospital, School of Medicine, Shanghai Jiao Tong University); Hanmin Liu , Lingli Zhang (West China Second University Hospital, Sichuan University)

**The development group:**

Dehui Chen (The First Affiliated Hospital of Guangzhou Medical University),

Lina Chen (West China Second Hospital, Sichuan University),

Xinxin Chen (Guangzhou Women and Children's Medical Center)

Xing Chen (Shandong Provincial Hospital Affiliated to Shandong First Medical University),

Zhimin Chen (Children's Hospital, Zhejiang University School of Medicine),

Xiaoyan Dong (Shanghai Children's Hospital, School of Medicine, Shanghai Jiao Tong University),

Liang Huang (West China Second Hospital, Sichuan University),

Yi Ji (West China Hospital, Sichuan University),

Yongmei Jiang (West China Second University Hospital, Sichuan University),

Zhiping Li (Children's Hospital of Fudan University),

Enmei Liu (Children's Hospital of Chongqing Medical University),

Shuhua Luo (West China Second Hospital, Sichuan University)

Guangmin Nong (The First Affiliated Hospital of Guangxi Medical University),

Yun Peng (Beijing Children's Hospital, Capital Medical University),

Suyun Qian (Beijing Children's Hospital, Capital Medical University),

Tianyou Wang (Beijing Children's Hospital, Capital Medical University),

Xinyu Yuan (The Affiliated Children's Hospital, Capital Institute of Pediatrics),

Hao Zhang (Shanghai Children's Medical Center, Shanghai Jiaotong University School of Medicine)

Linan Zeng (West China Second Hospital, Sichuan University),

Hong Zhang (Shanghai Children's Hospital, School of Medicine, Shanghai Jiao Tong University),

Xiaobo Zhang (Children’s Hospital of Fudan University),

Hailin Zhang (The Second Affiliated Hospital and Yuying Children's Hospital of Wenzhou Medical University),

Deyu Zhao (Children's Hospital of Nanjing Medical University),

Shunying Zhao (Beijing Children's Hospital, Capital Medical University),

Xiufang Zhao (West China Second Hospital, Sichuan University),

Kaiyu Zhou (West China Second Hospital, Sichuan University),

Yingxue Zou (Children's Hospital of Tianjin University).

**2. The secretary group:** Jianing Liu, Xiaofeng Ni, Xia Song, Yi Wu, Haiyang Zhang (West China Second Hospital of Sichuan University)

**Appendix 2.** **Summary of evidence**

**Table 1** Meta analysis of evidence of clinical manifestations in children with PTE

**Table 2** Summary of evidence of clinical likelihood rating score alone or combined D-dimer testing for the diagnosis of PTE

**Table 3** Meta analysis of evidence of D-dimer testing alone for the diagnosis in children with PTE

**Table 4** Summary of evidence of CT pulmonary angiography for the diagnosis in children with of PTE

**Table 5** Summary of evidence of magnetic resonance pulmonary arteriography for the diagnosis in children with of PTE

**Table 6** Summary of evidence of anticoagulation indications in children with symptomatic PTE

**Table 7** Summary of evidence of anticoagulation indications in children with asymptomatic PTE

**Table 8** Summary of evidence of LMWH vs other anticoagulants in children with PTE

**Table 9** Summary of evidence of LMWH vs VKA in children with PTE

**Table 10** Meta-analysis of evidence of combined antithrombin therapy vs standard anticoagulation therapy in children

**Table 11** Summary of evidence of anticoagulation courses in children with idiopathic PTE

**Table 12** Summary of evidence of anticoagulation courses in children with secondary PTE

**Table 13** Meta analysis of thrombolytic therapy vs anticoagulation only in children with VTE and/or PTE

**Table 14** Summary of evidence of catheter based therapy in children with PTE

**Table 15** Summary of evidence of interventional therapy in adults with PTE

**Table 16** Summary of evidence of indications in children with PTE who adopted surgical embolectomy

**Table 17** Summary of evidence of PERT establishment in patients with PTE

**Table 18** Summary of evidence of treatment of MP pneumonia complicated with PTE

**Table 19** Summary of evidence of venous filters palcement in children with PTE

**Table 20** Summary of Evidence of Pulmonary Thromboembolism Combined with Renal Impairment or Renal Failure

**Table 1** Meta analysis of evidence of clinical manifestations in children with PTE

| Population | Study characteristics | | Sample size | Results | Quality assessment | | | | | Quality |
| --- | --- | --- | --- | --- | --- | --- | --- | --- | --- | --- |
|  | № of studies | Study design |  |  | Risk of bias | Inconsistency | Indirectness | Imprecision | Publication bias |  |
| Children with PTE | 8[1-8] | Cross-section | 160 | Dyspnea: combined incidence 60% (95% CI 49%, 80%) | -1 | -1 | 0 | 0 | 0 | Very low |
|  | 8[1-3, 5, 7-10] | Cross-section | 75 | Cough: combined incidence 58% (95% CI 28%, 88%) | -1 | -1 | 0 | 0 | 0 | Very low |
|  | 10[1-3, 5-11] | Cross-section | 168 | Chest pain: combined incidence 56% (95% CI 47%, 64%) | -1 | 0 | 0 | -1 | 0 | Very low |
|  | 3[4, 6, 8] | Cross-section | 35 | Shortness of breath: combined incidence 46% (95% CI 6%, 98%) | -1 | -1 | 0 | 0 | 0 | Very low |
|  | 2[2, 5] | Cross-section | 19 | The typical triad: combined incidence 10% (95% CI 0%, 23%) | -1 | 0 | 0 | 0 | 0 | Very low |
|  | 6[3, 6-9, 11] | Cross-section | 33 | Hemoptysis: combined incidence 7% (95% CI 2%, 12%) | -1 | -1 | 0 | 0 | 0 | Very low |

*Notes:* PTE: pulmonary thromboembolism.

**Table 2** Summary of evidence of clinical likelihood rating score alone or combined D-dimer testing for the diagnosis in children with PTE

| Population | Study characteristics | | Sample size | Results | Quality assessment | | | | | Quality |
| --- | --- | --- | --- | --- | --- | --- | --- | --- | --- | --- |
|  | № of studies | Study design |  |  | Risk of bias | Inconsistency | Indirectness | Imprecision | Publication bias |  |
| Children with PTE | 1[8] | Cross-section | 48 | Adult PTE exclusion criteria: sensitivity 60%, specificity 46%  Pediatric PTE scoring model: sensitivity 50%, specificity 75%  Pediatric PTE scoring model/PTE exclusion criteria + D-dimer testing: no increase in specificity, sensitivity | 0 | 0 | 0 | -1 | 0 | Very low |
|  | 1[7] | Cross-section | 24 | Sensitivity of Wells score: 37.5% | -1 | 0 | 0 | -1 | 0 | Very low |
|  | 1[12] | Cross-section | 104 | Pediatric thrombosis decision rules: specificity 69%, sensitivity 69%, negative predictive value 98% | -1 | 0 | -1 | -1 | 0 | Very low |
| Children with VTE |  |  |  |  |  |  |  |  |  |  |
| Adults with suspected PE* | 1[13] | Cross-section | 965 | Wells score excludes adults with PE, which occurs in 12%-13% of cases | 0 | 0 | 0 | 0 | 0 | Low |
|  | 1[14] | Cross-section | 3306 | The incidence of PE was 0.3%-0.5% in adults with a Wells score ≤4 and normal D-dimers | 0 | 0 | 0 | 0 | 0 | Low |

*Notes:* PTE: pulmonary thromboembolism; VTE: Venous Thromboembolism; Adults with suspected PE*: As indirect evidence for children.

**Table 3** Meta analysis of evidence of D-dimer testing alone for the diagnosis in children with PTE

| Population | Study characteristics | | Sample size | Results | Quality assessment | | | | | Quality |
| --- | --- | --- | --- | --- | --- | --- | --- | --- | --- | --- |
|  | № of studies | Study design |  |  | Risk of bias | Inconsistency | Indirectness | Imprecision | Publication bias |  |
| Children with PE | 6[15-20] | Cross-section | 2052 | Sensitivity: 89.0% (95% CI 63.0%, 98.0%),  Specificity: 53.0% (95% CI 34.0%, 71.0%). | -1 | 0 | 0 | -1 | 0 | Very low |

*Notes:* PTE: pulmonary thromboembolism.

**Table 4** Summary of evidence of CT pulmonary angiography for the diagnosis in children with PTE

| Population | Study characteristics | | Sample size | Results | Quality assessment | | | | | Quality |
| --- | --- | --- | --- | --- | --- | --- | --- | --- | --- | --- |
|  | № of studies | Study design |  |  | Risk of bias | Inconsistency | Indirectness | Imprecision | Publication bias |  |
| Children with PTE | 1[21] | Cross-section | 113 | When ＜24 HU was used as the diagnostic threshold, the sensitivity was 93.9%, the specificity was 73.8%, the positive predictive value was 59.6%, and the negative predictive value was 96.7%;  When <22 HU was used as the diagnostic threshold, the sensitivity was 79.3%, the specificity was 82.1%, the positive predictive value was 57.5%, and the negative predictive was value 92.8%. | 0 | 0 | 0 | -1 | 0 | Very  Low |
|  | 1[22] | Cross-section | 9 | PTE diagnostic accuracy: 91.7%-100% (average 96.7%) | 0 | 0 | 0 | -1 | 0 | Very  Low |

*PTE: pulmonary thromboembolism

**Table 5** Summary of evidence of magnetic resonance pulmonary arteriography for the diagnosis in children with PTE

| Population | Study characteristics | | Sample size | Results | Quality assessment | | | | | Quality |
| --- | --- | --- | --- | --- | --- | --- | --- | --- | --- | --- |
|  | № of studies | Study design |  |  | Risk of bias | Inconsistency | Indirectness | Imprecision | Publication bias |  |
| Adults with PTE* | 1[23] | Cross-section | 104 | Sensitivity: 78% (95%CI 67%, 86%) specificity: 99% (95%CI 96%, 100%), positive predictive value: 78.8 (95%CI 19.7, 315), negative predictive value: 0.23 (95%CI 0.15, 0.34) | -1 | 0 | -1 | -1 | 0 | Very  Low |

*Notes:*PTE: pulmonary thromboembolism; Adults with PTE*: As indirect evidence for children.

**Table 6** Summary of evidence of anticoagulation indications in children with symptomatic PTE

| Population | Study characteristics | | Sample size | | Results | Quality assessment | | | | | Quality |
| --- | --- | --- | --- | --- | --- | --- | --- | --- | --- | --- | --- |
|  | № of studies | Study design | Anticoagulation | No anticoagulation |  | Risk of bias | Inconsistency | Indirectness | Imprecision | Publication bias |  |
| Children with intermediate to high-risk of PTE | 1[11] | Cross-section | 23 | 4 | Major bleedin^#^g:  Anticoagulation 7/23 (30.4%)  No anticoagulation 0/4 (0%)  relative risk (RR)：4.09 (95%CI 0.19, 86.09) | 0 | 0 | 0 | 0 | 0 | Low |
| Children with symptomatic DVT or PTE | 1[24] | Cross-section | 55 | - | Mortality^#^:2/55 (3.6%) | -1 | 0 | 0 | 0 | 0 | Very low |
|  | 40[25-30] | Cross-section | 1827 | - | Major bleeding: 0.9% (95%CI 0.0%, 5.9%) | -1 | 0 | 0 | 0 | 0 | Very low |
| Children with symptomatic or asymptomatic DVT or PTE | 3[26, 30, 31] | Cross-section | 267 | - | PTE exacerbation: 0.3% (95%CI 0.0, 2.1%) | -1 | 0 | -1 | 0 | 0 | Very low |
|  | 38[25-29] | Cross-section | 1590 | - | VTE recurrence: 1.7% (95%CI 1.0%, 2.5%) | -1 | 0 | 0 | 0 | 0 | Very low |
| Adults with PTE* | 1[32] | RCT(first stage, stop for the low mortality for anticoagulation group) | 16  Com | 19 | Mortality^#^:  Anticoagulation: 1/16 (6.3%)  No anticoagulation: 5/19 (26.3%)  relative risk(RR) 0.24 (95%CI 0.03, 1.83)  VTE recurrence^#^:  Anticoagulation: 0/16 (0%)  No anticoagulation: 5/19(26.3%)  RR 0.11 (95%CI 0.01, 1.80) | -1 | 0 | -1 | -1 | 0 | Very low |
|  | 1[32] | RCT(complete Series) | 54 | 19 | Deaths from PE^#^  Anticoagulation: 0/54 (0%)  No anticoagulation: 5/19 (26.3%)  RR 0.24 (95%CI 0.03, 1.83)  VTE recurrence^#^:  Anticoagulation:1/54 (1.85%)  No anticoagulation: 5/19(26.3%)  RR 0.07 (95%CI 0.01, 0.57) | -1 | 0 | -1 | -1 | 0 | Very low |

*Notes:* DVT: deep venous thrombosis; PTE: pulmonary thromboembolism; VTE: Venous Thromboembolism; Adults with suspected PE*: As indirect evidence for children; # number of events/number of patients (incidence)

**Table 7** Summary of evidence of anticoagulation indications in children with asymptomatic PTE

| Population | Study characteristics | | Sample size | | Results | Quality assessment | | | | | Quality |
| --- | --- | --- | --- | --- | --- | --- | --- | --- | --- | --- | --- |
|  | № of studies | Study design | Anticoagulation | No anticoagulation |  | Risk of bias | Inconsistency | Indirectness | Imprecision | Publication bias |  |
| Children with asymptomatic DVT or PTE | 1[33] | Cross-section | - | 31 | Mortality^#^: 0/31 (0%)  Thrombus extension or clinical embolism: 0/31 (0%)  Post-thrombotic syndrome (PTS): 1/31 (3.2%) | -1 | 0 | 0 | 0 | 0 | Very low |
| Children with asymptomatic or symptomatic DVT or PTE | 3[26, 30, 31] | Cross-section | 267 | - | PTE exacerbation: 0.3% ( 95%CI 0.0, 2.1%) | -1 | -1 | -1 | -1 | 0 | Very low |
|  | 3[26, 30, 31] | Cross-section | 267 | - | Major bleeding: 0.3% (95%CI 0.1%, 1.8%) | -1 | -1 | 0 | -1 | 0 | Very low |

*Notes:* DVT: deep venous thrombosis; PTE: pulmonary thromboembolism; PTS：Post-Thrombotic Syndrome; # number of events/number of patients (incidence)

**Table 8** Summary of evidence of LMWH vs other anticoagulants in children with PTE

| Population | Study characteristics | | Sample size | | Results | Quality assessment | | | | | Quality |
| --- | --- | --- | --- | --- | --- | --- | --- | --- | --- | --- | --- |
|  | № of studies | Study design | LMWH | Other |  | Risk of bias | Inconsistency | Indirectness | Imprecision | Publication bias |  |
| Children with DVT or PTE (without distinction whether if symptomatic  patients) | 1[34] | Case series | 10 | 38 (LMWH+thrombolytic) | Bleeding^#^:  LMWH: 0/10 (0%), Other: 2/38,(5.3%)  RR 0.71 (95%CI 0.04,13.71) | -1 | 0 | 0 | 0 | 0 | Very low |
|  | 1[35] | RCT | 36 | 40  (UFH/  warfarin) | VTE-related mortality^#^:  LMWH: 0/36(0%), Other: 0/40(0%)  RR Not estimable  Other mortality^#^:  LMWH: 1/36 (2.8%), Other: 4/40(10.0%)  RR 0.28 (95%CI 0.03,2.37)  PT^#^E:  LMWH: 0/36(0%), Other: 1/40(2.5%)  RR 0.37(95%CI 0.02,8.79)  DVT^#^:  LMWH: 2/36(5.6%), Other: 4/40(10.0%)  RR 0.56(0.11,2.85)  VTE^#^:  LMWH: 2/36(5.6%), Other: 5/40(12.5%)  RR 0.44 (95%CI 0.09,2.15)  Major bleeding^#^:  LMWH: 2/36(5.6%), Other: 5/40(12.5%)  RR 0.44 (95%CI 0.09,2.15)  Minor bleeding^#^:  LMWH: 32/36 (88.9%), Other: 27/40 (67.5%)  RR 1.32(95%CI 1.03,1.68)  Any bleeding^#^:  LMWH: 32/36 (88.9%), Other: 28/40(70.0%)  RR 1.27(95%CI 1.01,1.60) | -1 | 0 | 0 | -1 | 0 | Low |
| Adult with DVT or PTE* | 1[36] | RCT | 97 | 103  (UFH) | Newly occurring PTE^#^:  LMWH: 0/97 (0%), Other: 4/103(3.9%)  RR 0.11 (95%CI 0.01, 2.13) | 0 | 0 | -1 | -1 | 0 | Low |
|  | 1[37] | RCT | 1103 | 1110  (UFH) | VTE recurrence^#^：  LMWH: 42/1103 (3.8%), Other: 56/1110 (5.0%)  RR 0.75 (95%CI 0.49, 1.12)  Major bleeding^#^:  LMWH: 14/1103 (1.3%), Other: 12/1110 (1.1%)  RR 1.18 (95%CI 0.54, 2.55) | -1 | 0 | -1 | -1 | 0 | Very low |
|  | 1[38] | Cohort | 117 | 50(UFH) | Bleeding^#^:  LMWH: 11/117 (9.4%), Other: 5/50 (10.0%)  RR 0.93 (95%CI 0.31, 2.84)  Mortality^#^:  LMWH: 4/117 (3.4%), Other: 4/50 (8.0%)  RR 0.41 (95%CI 0.10, 1.70)  Total cost^#^：  LMWH: $69.11, Other: $88.39 | 0 | 0 | -1 | 0 | 0 | Very low |

*Notes:* DVT: deep venous thrombosis; PTE: pulmonary thromboembolism; VTE: Venous Thromboembolism; Adult with DVT or PTE*: As indirect evidence for children; # number of events/number of patients (incidence)

**Table 9** Summary of evidence of LMWH vs VKA in children with PTE

| Population | Study characteristics | | Sample size | | Results | Quality assessment | | | | | Quality |
| --- | --- | --- | --- | --- | --- | --- | --- | --- | --- | --- | --- |
|  | № of studies | Study design | LMWH | VKA |  | Risk of bias | Inconsistency | Indirectness | Imprecision | Publication bias |  |
| Children with DVT or PTE (without distinction whether if symptomatic  patients) | 1[35] | RCT | 36 | 40 | Mortality(follow up 6m)^#^:  LMWH: 1/36 (2.8%), VKA: 4/40 (10.0%)  RR 0.28 (95%CI 0.03, 2.37) | -1 | 0 | 0 | -1 | 0 | Low |
|  | 1[35] | RCT | 36 | 40 | PTE exacerbation(follow up 6m):  LMWH: 0/36 (0.0%), VKA: 1/40 (2.5%)  RR 0.37 (95%CI 0.01, 8.79) | -1 | 0 | 0 | -1 | 0 | Low |
|  | 2[26, 30] | Cross-section | - | 237 | PTE exacerbation^#^:  VKA: 0.3% (95%CI 0.0%, 1.7%) | -1 | 0 | 0 | 0 | 0 | Very low |
|  | 37[25, 27, 28] | Cross-section | 1483 | - | VTE recurrence^#^:  LMWH: 1.5% (95%CI 0.8%, 2.3%) | -1 | 0 | 0 | -1 | 0 | Very low |
|  | 1[29] | Cross-section | - | 107 | VTE recurrence^#^: VKA: 3/107 (2.8%) | -1 | 0 | 0 | 0 | 0 | Very low |
|  | 1[35] | RCT | 36 | 40 | Major bleeding(follow up 6m)^#^:  LMWH: 2/36 (5.6%), VKA: 5/40 (12.5%)  RR 0.44 (95%CI 0.09, 2.15) | -1 | 0 | 0 | -1 | 0 | Low |
|  | 37[25, 27, 28] | Cross-section | 1483 | - | Major bleeding^#^:  LMWH: 2.5% (95%CI 0.0%, 12.1%) | -1 | 0 | 0 | 0 | 0 | Very low |
|  | 22[26, 29, 30] | Cross-section | - | 344 | Major bleeding^#^:  VKA: 0.3% (95%CI 0.0%, 1.5%) | -1 | 0 | 0 | 0 | 0 | Very low |

*Notes:* DVT: deep venous thrombosis; PTE: pulmonary thromboembolism; VTE：Venous Thromboembolism; # number of events/number of patients (incidence)

**Table 10** Meta-analysis of evidence of combined antithrombin therapy vs standard anticoagulation therapy in children

| Population | Study characteristics | | Sample size | | Results | Quality assessment | | | | | Quality |
| --- | --- | --- | --- | --- | --- | --- | --- | --- | --- | --- | --- |
|  | № of studies | Study design | Combined antithrombin therapy | Standard anticoagulation therapy |  | Risk of bias | Inconsistency | Indirectness | Imprecision | Publication bias |  |
| Children with PTE | 1[39] | NA | NA | NA | Bleeding^a^:  Combined AT: 14.3%  Standard: 3.9%, *P* = 0.55 | Unable to assess due to not published | | | | | |
| Children treated with combined antithrombin vs. standard anticoagulation therapy | 2[40, 41] | RCT | 91 | 91 | Mortality^#^:  Combined AT: 8/91 (8.8%)  Standard: 5/91 (5.5%)  RR 1.49 (95%CI 0.38, 5.87) | -1 | 0 | -1 | -1 | 0 | Very low |
|  | 6[42-47] | Cohort | 2312 | 7048 | Mortality^#^:  Combined AT: 901/2312 (39.0%)  Standard: 2833/7048 (40.2%)  RR 0.98 (95%CI 0.68, 1.40) | -1 | 0 | 0 | -1 | 0 | Very low |
|  | 4[42, 44, 46, 47] | Cohort | 2212 | 6857 | DVT^#^:  Combined AT: 488/2212 (22.1%)  Standard: 1063/6857 (15.5%)  RR 1.35 (95%CI 0.97, 1.90) | 0 | 0 | -1 | -1 | 0 | Very low |
|  | 4[40, 41, 48, 49] | RCT | 124 | 160 | Major bleeding^#^:  Combined AT: 32/124 (25.8%)  Standard:25/160 (15.6%)  RR 1.25 (95%CI 0.74, 2.11) | -1 | 0 | -1 | -1 | 0 | Very low |
|  | 6[42, 43, 45-48] | Cohort | 2177 | 7026 | Major bleeding^#^:  Combined AT: 926/2177 (42.5%)  Standard: 2581/7026 (36.7%)  RR 1.23 (95%CI 1.16, 1.30) | -1 | 0 | -1 | 0 | 0 | Very low |

*Notes:* DVT: deep venous thrombosis; a: this study only reported the incidence; # number of events/number of patients (incidence)

**Table 11** Summary of evidence of anticoagulation courses in children with idiopathic PTE

| Population | Study characteristics | | Sample size | | Results | Quality assessment | | | | | Quality |
| --- | --- | --- | --- | --- | --- | --- | --- | --- | --- | --- | --- |
|  | № of studies | Study design | Anticoagulation>6m | Anticoagulation≤6m |  | Risk of bias | Inconsistency | Indirectness | Imprecision | Publication bias |  |
| Children with idiopathic DVT or PTE | 1[50] | Cohort | 56 | 27 | VTE recurrence(Enoxaparin)^#^:  Anticoagulation>6-12m: 13/56 (23.2%)  Anticoagulation3-6m: 4/27 (14.8%)  <3m: 14/66 (21%)  Odds Ratio(OR) 1.74 (95%CI 0.51, 5.95) | 0 | 0 | 0 | -1 | 0 | Very low |

*Notes:* DVT: deep venous thrombosis; PTE: pulmonary thromboembolism; VTE: Venous Thromboembolism; # number of events/number of patients (incidence)

**Table 12** Summary of evidence of anticoagulation courses in children with secondary PTE

| Population | Study characteristics | | Sample size | | Results | Quality assessment | | | | | Quality |
| --- | --- | --- | --- | --- | --- | --- | --- | --- | --- | --- | --- |
|  | № of studies | Study design | Anticoagulation>3m | Anticoagulation≤3m |  | Risk of bias | Inconsistency | Indirectness | Imprecision | Publication bias |  |
| Children with secondary DVT or PTE | 1[50] | Cohort | 83 | 66 | VTE recurrence(Enoxaparin)^#^:  Anticoagulation>3m: 17/83(20.5%)  Anticoagulation≤3m: 14/66(21.2%)  OR 1.74 (95%CI 0.51-5.95) | 0 | 0 | 0 | -1 | 0 | Very low |
|  | 1[35] | RCT | 76 | NR | VTE recurrence (follow up 6m, LWMH/VKA^#^):  Anticoagulation 3-6m：6/76 (7.9%)  Major bleeding (follow up 6m, LWMH/VKA)^#^:  Anticoagulation 3-6m：7/76 (9.2%)  Mortality (follow up 6m, LWMH/VKA)^#^:  Anticoagulation 3-6m：5/76 (6.6%) | 0 | 0 | -1 | -1 | 0 | Low |

*Notes:* DVT: deep venous thrombosis; PTE: pulmonary thromboembolism; VTE：Venous Thromboembolism; # number of events/number of patients (incidence)

**Table 13** Meta analysis of thrombolytic therapy vs anticoagulation only in children with VTE and/or PTE

| Population | Study characteristics | | Sample size | | Results | Quality assessment | | | | | Quality |
| --- | --- | --- | --- | --- | --- | --- | --- | --- | --- | --- | --- |
|  | № of studies | Study design | Thrombolytic therapy | Anticoagulation only or None |  | Risk of bias | Inconsistency | Indirectness | Imprecision | Publication bias |  |
| Children with high-risk PTE | 3 [11, 51, 52] | Cohort | 9 | 19 | All-cause hospital mortality^#^  Thrombolytic after anticoagulation: 3/9 (33.33%)  Anticoagulation only or None: 9/19 (47.37%)  RR 0.70 (95%CI 0.38, 1.29)  PE-related mortality^#^  Thrombolytic after anticoagulation: 2/9 (22.22%)  Anticoagulation only or None: 6/19 (31.58%)  RR 0.70 (95%CI 0.17, 2.89)  Fatal major bleeding^#^  Thrombolytic after anticoagulation: 0/9 (0%)  Anticoagulation only or None: 0/19 (0%)  RR Not estimable  Nonfatal major bleeding^#^  Thrombolytic after anticoagulation: 1/9 (11.11%)  Anticoagulation only or None: 6/19 (31.58%)  RR 0.35 (95%CI 0.05, 2.45)  CTEPH^#^  Thrombolytic after anticoagulation: 2/9 (22.22%)  Anticoagulation only or None: 0/19 (0%)  RR 5.11 (95%CI 0.28, 93.55) | 0 | 0 | 0 | 0 | 0 | Low |
| Children with intermediate -risk PTE | 3[11, 51, 52] | Cohort | 13 | 20 | All-cause hospital mortality^#^  Thrombolytic after anticoagulation: 0/13(0%)  Anticoagulation only or None: 4/20(20%)  RR 0.09 (95%CI 0.01, 1.66)  PE-related mortality^#^  Thrombolytic after anticoagulation: 0/13(0%)  Anticoagulation only or None: 2/20(10%)  RR 0.17 (95%CI 0.01, 3.26)  Fatal major bleeding^#^  Thrombolytic after anticoagulation: 0/13(0%)  Anticoagulation only or None: 1/2(50%)  RR 0.07 (95%CI 0.00, 1.15)  Nonfatal major bleeding^#^  Thrombolytic after anticoagulation: 0/13(0%)  Anticoagulation only or None: 0/20(0%)  RR Not estimable  CTEPH^#^  Thrombolytic after anticoagulation: 0/13(0%)  Anticoagulation only or None: 2/20(10%)  RR 0.17 (95%CI 0.01, 3.26) | 0 | 0 | 0 | 0 | 0 | Low |
| Children with undifferentiated-risk PTE | 1[24] | Cross-section | 8 | 48 | Major bleeding^#^:  Thrombolysis with anticoagulation: 4/8 (50%)  Anticoagulation only or None: 8/48 (16.7%)  RR 3.00 (1.17, 7.67) | 0 | 0 | 0 | 0 | 0 | Low |
| Children with embolism  not differentiated by location | 1[53] | Cohort | 9 | 13 | All cause mortality^#^:  Thrombolytic therapy: 0/9 (0%)  Anticoagulation only or None: 0/13 (0%)  RR Not estimable  DVT^#^:  Thrombolytic therapy: 0/9 (0%)  Anticoagulation only or None: 0/13 (0%)  RR Not estimable  Major bleeding^#^:  Thrombolytic therapy: 1/9 (11.11%)  Anticoagulation only or None: 0/13(0%)  RR 3.67 (95%CI 0.16, 83.79)  PTS^#^  Thrombolytic therapy: 1/9 (11.11%)  Anticoagulation only or None: 8/13 (61.54%)  RR 0.18 (95%CI 0.03, 1.25) | -1 | 0 | 0 | -1 | 0 | Very low |
|  | 11[54-64] | Cohort | 275 | - | All cause mortality^#^:  0.7% (95%CI 0.0%, 3.3%)  Major bleeding^#^:  1.3% (95%CI 0.0%, 3.6%) | -1 | 0 | 0 | -1 | 0 | Very low |
|  | 12[54-65] | Cohort | 302 | - | DVT^#^:  25.2% (95%CI 8.4%, 46.5%) | -1 | 0 | 0 | -1 | 0 | Very low |
|  | 3[61, 63, 64] | Cohort | 101 | - | PTS^#^:  28.1 (95%CI 2.9%, 64.1%) | -1 | 0 | 0 | -1 | 0 | Very low |
|  | 2[24, 66] | Cross-section | 14 | - | Complete regression of thrombus: 6  Partial regression of thrombus: 5  No regression of thrombus: 3  Haemorrhage: 7  Fatal haemorrhage: 0  Death: 3  Pulmonary hypertension: 1 | -1 | 0 | 0 | 0 | 0 | Very low |

*Notes:* DVT: deep venous thrombosis; PTE: pulmonary thromboembolism; VTE：Venous Thromboembolism; # number of events/number of patients

**Table 14** Summary of evidence of catheter based therapy in children with PTE

| Population | Study characteristics | | Sample size | | Results | Quality assessment | | | | | Quality |
| --- | --- | --- | --- | --- | --- | --- | --- | --- | --- | --- | --- |
|  | № of studies | Study design | Catheter-directed thrombolysis | Systemic Thrombolysis |  | Risk of bias | Inconsistency | Indirectness | Imprecision | Publication bias |  |
| Children with high-risk PTE | 3[11, 51, 52] | Cohort | 5 | 9 | All-cause hospital mortality^#^  Catheter-directed thrombolysis: 2/5 (40.00%)  Systemic Thrombolysis: 3/9 (33.3%)  RR 1.20 (95%CI 0.28, 5.15)  PE-related mortality^#^  Catheter-directed thrombolysis: 1/5 (20.00%)  Systemic Thrombolysis: 2/9 (22.2%)  RR 0.90 (95%CI 0.10, 8.23)  Fatal major bleeding^#^  Catheter-directed thrombolysis: 0/2 (0%)  Systemic Thrombolysis: 0/9 (0%)  RR Not estimable  Nonfatal major bleeding^#^  Catheter-directed thrombolysis: 0/5 (0%)  Systemic Thrombolysis: 1/9 (11.1%)  RR 0.00 (95%CI 0.00, 3.07)  CTEPH^#^  Catheter-directed thrombolysis: 0/5 (0%)  Systemic Thrombolysis: 2/9 (22.2%)  RR 0.00 (95%CI 0.00, 1.53) | 0 | 0 | 0 | 0 | 0 | Low |
| Children with intermediate-risk PTE | 3[11, 51, 52] | Cohort | 2 | 13 | All-cause hospital mortality^#^  Catheter-directed thrombolysis: 0/2 (0%)  Systemic Thrombolysis: 0/13 (0%)  RR Not estimable  PE-related mortality^#^  Catheter-directed thrombolysis: 0/2 (0%)  Systemic Thrombolysis: 0/13 (0%)  RR Not estimable  Fatal major bleeding^#^  Catheter-directed thrombolysis: 0/2 (0%)  Systemic Thrombolysis: 0/13 (0%)  RR Not estimable  Nonfatal major bleeding^#^  Catheter-directed thrombolysis: 0/2 (0%)  Systemic Thrombolysis: 0/13 (0%)  RR Not estimable  CTEPH^#^  Catheter-directed thrombolysis: 0/2 (0%)  Systemic Thrombolysis: 0/13 (0%)  RR Not estimable | 0 | 0 | 0 | 0 | 0 | Low |
|  | 1[67] | Case series | 5 | - | Complete regression of thrombus: 4  Partial regression of thrombus: 1  Haemorrhage/clinically related non-haemorrhage: 0  CTEPH: 0 | -1 | 0 | 0 | 0 | 0 | Very low |
| Children with intermediate to high-risk PTE | 1[67] | Cross-section | 6 | - | Complete remission within 24 hours: 4  Partial remission within 24 hours: 2  Right ventricular function improvement: 6  Death: 0  Treatment-related complications: 0 | -1 | 0 | 0 | 0 | 0 | Very low |
| Children with PTE | 1[68] | Cross-section | 9 | - | Clinical improvement (improvement in cardiopulmonary function): 7/9  Complete resolution of thrombus: 4  Partial resolution of thrombus: 5  Bleeding complications: 0  Mean pulmonary artery pressure decreased in all (before: 37±11mm Hg; after: 28±10mm Hg, P = 0.0164) | -1 | 0 | 0 | 0 | 0 | Very low |

*Notes:* CTEPH: Chronic Thromboembolic Pulmonary Hypertension; PTE: pulmonary thromboembolism; # number of events/number of patients

**Table 15** Summary of evidence of interventional therapy in adults with PTE

| Population | Study characteristics | | Sample size | | Results | Quality assessment | | | | | Quality |
| --- | --- | --- | --- | --- | --- | --- | --- | --- | --- | --- | --- |
|  | № of studies | Study design | Catheter-directed thrombolysis | Others |  | Risk of bias | Inconsistency | Indirectness | Imprecision | Publication bias |  |
| Adults with PTE* | 9[69-71] | Cross-section | 448 | - | Mortality: 2.7%~3.6%  Mean pulmonary artery pressure:  baseline: 51.4 mm Hg  Postoperative: 36.9 mm Hg, p < 0.0001  Mean RV/LV: baseline: 1.36-1.55  Postoperative: 1.03-1.13, p < 0.0001  Obstruction index: baseline: 22.5s  Postoperative: 15.8, p < 0.0001  Improvement in right heart strain: 89.1%  Surgery-related major complications: 0%  Surgery-related minor bleeding: 10.7%  Bleeding complications: 3.6%  Major/fatal bleeding: 0%  Haemorrhagic stroke: 0% | -1 | 0 | -1 | 0 | 0 | Very low |
|  | 1[72] | Meta analysis | 860 | NR | Pulmonary artery pressure decreased:  Mean15 mm Hg (95%CI 11, 19)  Decrease in RV/LV ratio:  Mean 0.34 (95%CI 0.25, 0.42)  Cerebral haemorrhage: 3/860 (0.4%)  Major vascular complications: 40/860 (4.6%)  Mortality: 29/860 (3.4%) | -1 | 0 | -1 | 0 | 0 | Very low |
| Adults with high-risk PTE* | 1[73] | RCT | 30 | 29  (UFH) | Right heart strain improved(RV/LV, Baseline vs. After 24h):  Catheter directed thrombolysis: 1.28±0.19 vs. 0.99±0.17, P<0.001  UFH: 1.20±0.14 vs. 1.17±0.20, P=0.31  Major haemorrhage: 0%  Minor haemorrhage^#^:  Catheter directed thrombolysis: 3/30 (10%)  UFH: 1/29 (3.4%)  RR 2.90 (95%CI 0.32, 26.30) | -1 | 0 | -1 | 0 | 0 | Low |

*Notes:* PTE: pulmonary thromboembolism; RV/;V: Right Ventricle/Left Ventricle; Adults with PTE*: As indirect evidence for children; Adults with high-risk PTE*: As indirect evidence for children; # number of events/number of patients

Table 16 Summary of evidence of indications in children with PTE who adopted surgical embolectomy

| Population | Study characteristics | | Sample size | | Results | Quality assessment | | | | | Quality |
| --- | --- | --- | --- | --- | --- | --- | --- | --- | --- | --- | --- |
|  | № of studies | Study design | Surgical | № of children |  | Risk of bias | Inconsistency | Indirectness | Imprecision | Publication bias |  |
| Children with PTE who adopted surgical embolectomy | 4[74-77] | Case series | 7 | 8 | PTE after palliative surgery (Fontan procedure): 4  tumorous embolism: 1  Failure of anticoagulant or thrombolytic therapy: 1  Premature infant with high risk of intracranial hemorrhage: 1 | -1 | 0 | 0 | 0 | 0 | Very low |
|  | 1[78] | Case control | 1 | 5 | Failure of anticoagulant or thrombolytic therapy | -1 | 0 | 0 | 0 | 0 | Very low |
|  | 2[24, 51] | Cohort | 7 | 87 | Tumorous embolism: 5  Intracranial hemorrhag: 2 | -1 | 0 | 0 | 0 | 0 | Very low |

*Notes:* PTE: pulmonary thromboembolism

**Table 17** Summary of evidence of PERT establishment in patients with PTE

| Population | Study characteristics | | Sample size | | Results | Quality assessment | | | | | Quality |
| --- | --- | --- | --- | --- | --- | --- | --- | --- | --- | --- | --- |
|  | № of studies | Study design | Before | After |  | Risk of bias | Inconsistency | Indirectness | Imprecision | Publication bias |  |
| Children with PTE | 1[79] | Cohort | 30 | 31 | Proportion of UFH^a^  Before: 90%  After: 95%  Proportion of Eligible and received reperfusion^#^  Before: 3/8 (38%)  After: 5/6 (83%), RR 2.18 (95%CI 0.85, 5.59), *P*<0.01  Proportion of Embolectomy/Thrombectomy^#^  Before: 0/30 (0%)  After: 1/31 (3%)  Proportion of catheter-directed thrombolysis^#^  Before: 3/30 (10%)  After: 3/31 (10%), RR 1.03 (95%CI 0.24, 4.44)  Proportion of Systemic TPA^#^  Before: 0/30 (0%)  After: 3/31 (10%)  Proportion of Reperfusion therapies^#^  Before: 3/30 (10%)  After: 5/31 (16%), RR 1.63 (95%CI 0.45, 5.91)  Proportion of LMWH^a#^  Before: 10%  After: 5%  Proportion of ECMO^#^  Before: 1/30 (3%)  After: 1/31 (3%), RR 1.03 (95%CI 0.07, 15.89)  Time-to-reperfusion  Before: 720min  After: 525min, *P*=0.10  Time-to-echocardiogram  Before: 282min  After: 120min, *P*=0.0147  Time-to-anticoagulation given  Before: 154min  After: 113min, *P*=0.049  Time-to-anticoagulation order  Before: 90min  After: 54min, *P*<0.01  Propotion of Major bleeding^#^  Before: 5/30 (17%)  After: 2/31 (6%), RR 0.36 (95%CI 0.07, 1.81)  Propotion of Clinically relevant nonmajor bleeding^#^  Before: 2/30 (7%)  After: 2/31 (6%), RR 0.97 (95%CI 0.15, 6.31)  Propotion of PE-related mortality^#^  Before: 2/30 (7%)  After: 1/31 (3%), RR 0.48 (95%CI 0.05, 4.76)  Stay in hospital  Before: 6 [IQR, 3-15] days  After: 6 [IQR, 4-17] days, *P*=0.72  Stay in ICU  Before: 1.5 [IQR, 0-3] days  After: 1 [IQR, 0-4.5] days, *P*=0.63 | 0 | 0 | 0 | 0 | 0 | Low |
| Adults with PTE* | 1[80] | Cohort | 212 | 228 | Proportion of CDL^#^:  Before: 10/212 (4.7%)  After: 31/212 (14.6%), RR 0.35(95%CI 0.17,0.69)  Proportion of advanced treatment^#^:  Before: 19/212 (8.9%)  After: 44/212 (20.7%), RR 0.46 (95%CI 0.28,0.77)  Major bleeding(follow up for 30 days)^#^:  Before: 6/118 (5.1%)  After: 13/228 (5.7%), RR 0.89 (95%CI 0.35,2.29)  Mortality^#^:  Before: 6/118 (5.1%)  After: 19/228 (8.3%), RR 0.61 (95%CI 0.25,1.49) | -1 | 0 | -1 | 0 | 0 | Very low |
|  | 1[81] | Cohort | 154 | 167 | Stay in ICU^#^:  Before: median 5 days  After: median 2 days, p < 0.01  Reperfusion rates^#^:  Before: 46/154 (29.9%)  After: 154/167 (92.2%)，RR 0.32 (95%CI 0.25,0.41)  Diagnosis-to-reperfusion time:  Before: 763min  After: 181min, 92% of included children，p < 0.01  No increase in bleeding complications  Overall survival at discharge remained unchanged | Unable to assess due to no full text | | | | | |
|  | 1[82] | Cohort | 159 | 146 | Change in treatment:  Before: UFH 85%+advanced treatment 15%  After: UFH 68%+advanced treatment 32%  Triage-to-diagnosis time:  Before: 384 min  After: 212min, *P* = 0.0001  Diagnosis-to-treatment time:  Before: 182 min  After: 76 min, *P* = 0.0001  Triage-to-treatments time:  Before: 392 min  After: 290min, *P* < 0.0001 | 0 | 0 | -1 | 0 | 0 | Very low |
|  | 1[83] | Cohort | 992 | 77 | Stay in hospital:  Before: 9.22±16.09  After: 6.31±7.44，RR 2.91 (95%CI 0.97,4.85)  Stay in ICU^b^:  Before: 6.86± 9.35 (351 of children)  After: 4.4±5.05 (48 of children), RR :2.46 (95%CI 0.73, 4.19)   1. day re-hospitalisation risk^a^:   Before: 0.91%  After: 0%, RR 1.49 (95%CI 0.09,25.40)  Mortality:  Before: 13.38%  After: 15.07%, RR 0.86 (95%CI 0.50, 1.48) | 0 | 0 | -1 | 0 | 0 | Very low |
|  | 1[84] | Cohort | 343 | 426 | Time to initiate anticoagulation:  Before: 16.3±23.3  After: 12.6±14.9, RR 3.70 (95%CI 0.86,6.54)  Significant/clinically relevant non-major bleeding:  Before: 54/318 (16.9%)  After: 34/408 (8.3%), RR 2.04 (95%CI 1.36 3.05)  Mortality rate 30 days or hospitalisation:  Before: 29/343 (8.4%),  After: 20/426 (4.7%), RR 1.80 (95%CI 1.04, 3.13) | -1 | 0 | -1 | 0 | 0 | Very low |

*Notes:* PTE: pulmonary thromboembolism. ^a^ The study did not report the number of children; b The study only included 351 children before PERT and 48 children after PERT for stay in ICU; Adults with PTE*: As indirect evidence for children; # number of events/number of patients

**Table 18** Summary of evidence of treatment of MP pneumonia complicated with PTE

| Population | Study characteristics | | Sample size | Treatment plan | Results | Quality assessment | | | | | Quality |
| --- | --- | --- | --- | --- | --- | --- | --- | --- | --- | --- | --- |
|  | № of studies | Study design |  |  |  | Risk of bias | Inconsistency | Indirectness | Imprecision | Publication bias |  |
| MP pneumonia complicated with PTE in children | 1[4] | Case series | 9 | LMWH+Warfarin | Discharged with improvement: 9  Embolus basically disappeared in imaging examination (follow up 1~3 months): 8 | 0 | 0 | 0 | -1 | 0 | Very low |
|  | 1[85] | Case series | 9 | Sequential LMWH with rivaroxaban;  2 of them with concomitant thrombolysis | Follow-up showed slow resorption, no thrombus recurrence, and normalization of autoantibody and thrombophilia markers:9 | 0 | 0 | 0 | -1 | 0 | Very low |
|  | 1[86] | Case series | 7 | LMWH | Improved and discharged: 2  No improvement and surgical treatment: 5 (deaths: 2) | 0 | 0 | 0 | -1 | 0 | Very low |
|  | 1[87] | Case series | 8 | LMWH+Aspirin | Discharged with improvement: 8  Embolus basically disappeared in imaging examination (follow up 1~3 months): 8 | 0 | 0 | 0 | -1 | 0 | Very low |
|  |  |  | 4 | LMWH or sodium sulfadiazepoxide | Embolus basically disappeared in imaging examination (follow up 1~3 months): 4 | 0 | 0 | 0 | -1 | 0 | Very low |
|  | 1[88] | Case series | 10 | LMWH + Rivaroxaban | Discharged with improvement: 10  Embolus basically disappeared in imaging examination (follow up 1~3 months): 10 | 0 | 0 | 0 | -1 | 0 | Very low |

*Notes:* PTE: pulmonary thromboembolism.MP: Mycoplasma pneumoniae

**Table 19** Summary of evidence of venous filters palcement in children with PTE

| Population | Study characteristics | | Sample size | | Results | Quality assessment | | | | | Quality |
| --- | --- | --- | --- | --- | --- | --- | --- | --- | --- | --- | --- |
|  | № of studies | Study design | Venous filter | Anticoagulation |  | Risk of bias | Inconsistency | Indirectness | Imprecision | Publication bias |  |
| Children with PTE | 1[24] | Cross-section | 7 | NA | Proximal extension of thrombus: 0%  Inability to remove venous filters due to adherent thrombus: 14.3%  Bleeding after initiation of anticoagulation: 0% | 0 | 0 | 0 | 0 |  | Low |
| Children with DVT or PTE | 7[89-95] | Cohort | 424 | NR | Mortality: 0.8% (0.0%, 4.7%) | -1 | 0 | -1 | -1 |  | Very low |
|  | 4[89, 90, 94, 95] | Cross-section | 102 | NA | Major bleeding: 0% | -1 | 0 | 0 | -1 |  | Very low |
|  | 6[89-92, 94, 95] | Cross-section | 148 | NA | Venous thrombosis due to IVC filters: 0.7% (95%CI 0.0%, 3.7%)  IVC filters malfunction: 1.7% (95%CI 0.0%, 7.1%) | -1 | 0 | 0 | -1 |  | Very low |

*Notes:* DVT: deep venous thrombosis; PTE: pulmonary thromboembolism; IVC: inferior vena cava.

**Table 20** Summary of Evidence of Pulmonary Thromboembolism Combined with Renal Impairment or Renal Failure

| Population | Study characteristics | | Sample size | Treatment plan | Results | Quality assessment | | | |  | Quality |
| --- | --- | --- | --- | --- | --- | --- | --- | --- | --- | --- | --- |
|  | № of studies | Study design |  |  |  | Risk of bias | Inconsistency | Indirectness | Imprecision | Publication bias |  |
| Children with nephrotic syndrome combined with PTE | 1[96] | Cross-section | 7 | UFH or LMWH standard anticoagulation bridging warfarin | Effectiveness: complete remission: 6 of 7 cases  Safety: death 0 of 7 cases; pulmonary hypertension 1 of 7 cases | 0 | 0 | 0 | -1 | 0 | Very low |
|  | 1[97] | Cross-section | 9 | Sequential UFH (4-5 days) bridging warfarin (6-12 months) | Effectiveness: full recovery 7 of 9 cases  Safety: death 2 of 9 cases | 0 | 0 | 0 | -1 | 0 | Very low |

*Notes:* PTE: pulmonary thromboembolism

**Reference:**

1. Agha BS, Sturm JJ, Simon HK, Hirsh DA. Pulmonary embolism in the pediatric emergency department. Pediatrics. 2013;132:663-7. doi:10.1542/peds.2013-0126.

2. Jingran M, Hongmei S, Juan X, Xiaoyan T, Yanyan H, Min W. Clinical analysis of 12 pediatric cases of antiphospholipid syndrome complicated with pulmonary embolism. Chinese Journal of Pediatrics. 2017;55:25-29.

3. Yinlan L, Zhuxia L, Jing T, Xinke D, Chang S. Clinical analysis of 8 cases with pulmonary embolism in children. Chinese Journal of Applied Clinical Pediatrics. 2020;35:852–55.

4. Lele K, Songlin Z, Bin Z, Lifang S, Shengli S. Features of the Morphological Distribution and Outcome Analysis of 9 Cases of Concurrent Pulmonary Artery Embolism of Mycoplasma Pneumonia in Children. Journal of Clinical Radiology. 2021;41:1331-35.

5. Xiao L, Lili D, Yu T, Min L, Jing W. Clinical analysis of mycoplasma pneumoniae pneumonia complicated with pulmonary thromboembolism. Chinese Clinical Medicine. 2021;28:328–32.

6. Fengqin L, Jing Z, Xing C, Ning D, Fangfang D, Ke W, et al. Clinical analysis and etiology factors of pulmonary embolism in 30 children. Chinese Journal of Applied Clinical Pediatrics. 2022;37:1386-91.

7. Hangül M, Köse M, Pekcan S, Çalışkan Ü, Tokgöz H, Aslan AT, et al. Pulmonary Embolism in Childhood: A Multicenter Experience from Turkey. Balkan medical journal. 2022;39:366-73. doi:10.4274/balkanmedj.galenos.2022.2022-3-46.

8. Ergenekon AP, Yilmaz Yegit C, Cenk M, Gulieva A, Kalyoncu M, Selcuk M, et al. The utility of risk assessment tools for acute pulmonary embolism in children. Pediatric pulmonology. 2023;58:55-60. doi:10.1002/ppul.26155.

9. Ke Y, Bin J, Pengjun Y, Jun Z. Clinical and imaging characteristics of pulmonary embolism complicating Mycoplasma pneumoniae pneumonia. Physician Online. 2022;12:7–10.

10. Lanqin C, Ju Y, Baoping X, Xiaomin D, Runhui W, Kunling S. Clinical manifestations and genetic analysis of 5 cases with inherited thrombophilia. Chinese Journal of Applied Clinical Pediatrics. 2022;37:934–38.

11. Pelland-Marcotte MC, Tucker C, Klaassen A, Avila ML, Amid A, Amiri N, et al. Outcomes and risk factors of massive and submassive pulmonary embolism in children: a retrospective cohort study. The Lancet Haematology. 2019;6:e144-e53. doi:10.1016/s2352-3026(18)30224-2.

12. Schultz RF, Sharathkumar A, Kwon S, Doerfer K, Lales G, Bhat R. Implementation of automatic data extraction from an enterprise database warehouse (EDW) for validating pediatric VTE decision rule: a prospective observational study in a critical care population. Journal of thrombosis and thrombolysis. 2020;50:782-89. doi:10.1007/s11239-020-02158-9.

13. Douma RA, Gibson NS, Gerdes VE, Büller HR, Wells PS, Perrier A, et al. Validity and clinical utility of the simplified Wells rule for assessing clinical probability for the exclusion of pulmonary embolism. Thrombosis and haemostasis. 2009;101:197-200.

14. Gibson NS, Sohne M, Kruip MJ, Tick LW, Gerdes VE, Bossuyt PM, et al. Further validation and simplification of the Wells clinical decision rule in pulmonary embolism. Thrombosis and haemostasis. 2008;99:229-34. doi:10.1160/th07-05-0321.

15. Biss TT, Brandão LR, Kahr WH, Chan AK, Williams S. Clinical probability score and D-dimer estimation lack utility in the diagnosis of childhood pulmonary embolism. Journal of thrombosis and haemostasis : JTH. 2009;7:1633-8. doi:10.1111/j.1538-7836.2009.03572.x.

16. Hennelly KE, Baskin MN, Monuteuax MC, Hudgins J, Kua E, Commeree A, et al. Detection of Pulmonary Embolism in High-Risk Children. The Journal of pediatrics. 2016;178:214-18.e3. doi:10.1016/j.jpeds.2016.07.046.

17. Kanis J, Hall CL, Pike J, Kline JA. Diagnostic accuracy of the D-dimer in children. Archives of disease in childhood. 2018;103:832-34. doi:10.1136/archdischild-2017-313315.

18. Kanis J, Pike J, Hall CL, Kline JA. Clinical characteristics of children evaluated for suspected pulmonary embolism with D-dimer testing. Archives of disease in childhood. 2018;103:835-40. doi:10.1136/archdischild-2017-313317.

19. Sharaf N, Sharaf VB, Mace SE, Nowacki AS, Stoller JK, Carl JC. D-dimer in Adolescent Pulmonary Embolism. Academic emergency medicine : official journal of the Society for Academic Emergency Medicine. 2018;25:1235-41. doi:10.1111/acem.13517.

20. Wang CX. Clinical prediction score in the diagnosis and evaluation of pulmonary embolism in children. In: Jilin University, 2023.

21. Sueyoshi E, Tsutsui S, Hayashida T, Ashizawa K, Sakamoto I, Uetani M. Quantification of lung perfusion blood volume (lung PBV) by dual-energy CT in patients with and without pulmonary embolism: preliminary results. European journal of radiology. 2011;80:e505-9. doi:10.1016/j.ejrad.2010.10.011.

22. Lee EY, Zucker EJ, Tsai J, Tracy DA, Cleveland RH, Zurakowski D, et al. Pulmonary MDCT angiography: value of multiplanar reformatted images in detecting pulmonary embolism in children. Ajr American Journal of Roentgenology. 2011;197:1460-5.

23. Stein PD, Chenevert TL, Fowler SE, Goodman LR, Gottschalk A, Hales CA, et al. Gadolinium-enhanced magnetic resonance angiography for pulmonary embolism: a multicenter prospective study (PIOPED III). Annals of internal medicine. 2010;152:434-43, w142-3. doi:10.7326/0003-4819-152-7-201004060-00008.

24. Biss TT, Brandão LR, Kahr WH, Chan AK, Williams S. Clinical features and outcome of pulmonary embolism in children. British journal of haematology. 2008;142:808-18. doi:10.1111/j.1365-2141.2008.07243.x.

25. Bidlingmaier C, Kenet G, Kurnik K, Mathew P, Manner D, Mitchell L, et al. Safety and efficacy of low molecular weight heparins in children: a systematic review of the literature and meta-analysis of single-arm studies. Seminars in thrombosis and hemostasis. 2011;37:814-25. doi:10.1055/s-0031-1297173.

26. Bonduel M, Sciuccati G, Hepner M, Torres AF, Pieroni G, Frontroth JP, et al. Acenocoumarol therapy in pediatric patients. Journal of thrombosis and haemostasis : JTH. 2003;1:1740-3. doi:10.1046/j.1538-7836.2003.00256.x.

27. Fiamoli V, Blatny J, Zapletal O, Kohlerova S, Janousova E. Treatment of Deep Vein Thrombosis with Continuous IV Infusion of LMWH: A Retrospective Study in 32 Children. Thrombosis. 2011;2011:981497. doi:10.1155/2011/981497.

28. O'Brien SH, Kulkarni R, Wallace A, Hamblin F, Burr S, Goldenberg NA. Multicenter dose-finding and efficacy and safety outcomes in neonates and children treated with dalteparin for acute venous thromboembolism. Journal of thrombosis and haemostasis : JTH. 2014;12:1822-5. doi:10.1111/jth.12716.

29. Spoor N, Smiers FJ, van der Meer FJ, Hutten BA, van Ommen CH. Phenprocoumon and acenocoumarol treatment in paediatric patients. Thrombosis and haemostasis. 2012;108:1238-41. doi:10.1160/th12-04-0242.

30. Streif W, Andrew M, Marzinotto V, Massicotte P, Chan AK, Julian JA, et al. Analysis of warfarin therapy in pediatric patients: A prospective cohort study of 319 patients. Blood. 1999;94:3007-14.

31. Andrew M, Marzinotto V, Massicotte P, Blanchette V, Ginsberg J, Brill-Edwards P, et al. Heparin therapy in pediatric patients: a prospective cohort study. Pediatric research. 1994;35:78-83. doi:10.1203/00006450-199401000-00016.

32. Barritt DW, Jordan SC. Anticoagulant drugs in the treatment of pulmonary embolism. A controlled trial. Lancet (London, England). 1960;1:1309-12. doi:10.1016/s0140-6736(60)92299-6.

33. Jones SE, Newall F, Monagle P, Cain T, Griffiths T, Butt W. Asymptomatic central venous catheter related thrombosis in children: two year follow up. Australian Critical Care. 2017;30:110. doi:<https://doi.org/10.1016/j.aucc.2017.02.005>.

34. Nohe N, Flemmer A, Rümler R, Praun M, Auberger K. The low molecular weight heparin dalteparin for prophylaxis and therapy of thrombosis in childhood: a report on 48 cases. European journal of pediatrics. 1999;158 Suppl 3:S134-9. doi:10.1007/pl00014339.

35. Massicotte P, Julian JA, Gent M, Shields K, Marzinotto V, Szechtman B, et al. An open-label randomized controlled trial of low molecular weight heparin compared to heparin and coumadin for the treatment of venous thromboembolic events in children: the REVIVE trial. Thrombosis research. 2003;109:85-92. doi:10.1016/s0049-3848(03)00059-8.

36. Hull RD, Raskob GE, Brant RF, Pineo GF, Elliott G, Stein PD, et al. Low-molecular-weight heparin vs heparin in the treatment of patients with pulmonary embolism. American-Canadian Thrombosis Study Group. Arch Intern Med. 2000;160:229-36. doi:10.1001/archinte.160.2.229.

37. Büller HR, Davidson BL, Decousus H, Gallus A, Gent M, Piovella F, et al. Subcutaneous fondaparinux versus intravenous unfractionated heparin in the initial treatment of pulmonary embolism. N Engl J Med. 2003;349:1695-702. doi:10.1056/NEJMoa035451.

38. Argenta C, Ferreira MA, Sander GB, Moreira LB. Short-term therapy with enoxaparin or unfractionated heparin for venous thromboembolism in hospitalized patients: utilization study and cost-minimization analysis. Value Health. 2011;14:S89-92. doi:10.1016/j.jval.2011.05.017.

39. Monagle P, Cuello CA, Augustine C, Bonduel M, Brandão LR, Capman T, et al. American Society of Hematology 2018 Guidelines for management of venous thromboembolism: treatment of pediatric venous thromboembolism. Blood advances. 2018;2:3292-316. doi:10.1182/bloodadvances.2018024786.

40. Schmidt B, Gillie P, Mitchell L, Andrew M, Caco C, Roberts R. A placebo-controlled randomized trial of antithrombin therapy in neonatal respiratory distress syndrome. American journal of respiratory and critical care medicine. 1998;158:470-6. doi:10.1164/ajrccm.158.2.9712116.

41. Fulia F, Cordaro S, Meo P, Gitto P, Gitto E, Trimarchi G, et al. Can the administration of antithrombin III decrease the risk of cerebral hemorrhage in premature infants? Biology of the neonate. 2003;83:1-5. doi:10.1159/000067005.

42. Haussmann U, Fischer J, Eber S, Scherer F, Seger R, Gungor T. Hepatic veno-occlusive disease in pediatric stem cell transplantation: impact of pre-emptive antithrombin III replacement and combined antithrombin III/defibrotide therapy. Haematologica. 2006;91:795-800.

43. Niebler RA, Christensen M, Berens R, Wellner H, Mikhailov T, Tweddell JS. Antithrombin replacement during extracorporeal membrane oxygenation. Artificial organs. 2011;35:1024-8. doi:10.1111/j.1525-1594.2011.01384.x.

44. Petäjä J, Peltola K, Rautiainen P. Disappearance of symptomatic venous thrombosis after neonatal cardiac operations during antithrombin III substitution. The Journal of thoracic and cardiovascular surgery. 1999;118:955-6; discussion 57. doi:10.1016/s0022-5223(99)70068-1.

45. Stansfield BK, Wise L, Ham PB, 3rd, Patel P, Parman M, Jin C, et al. Outcomes following routine antithrombin III replacement during neonatal extracorporeal membrane oxygenation. Journal of pediatric surgery. 2017;52:609-13. doi:10.1016/j.jpedsurg.2016.10.047.

46. Wong TE, Delaney M, Gernsheimer T, Matthews DC, Brogan TV, Mazor R, et al. Antithrombin concentrates use in children on extracorporeal membrane oxygenation: a retrospective cohort study. Pediatric critical care medicine : a journal of the Society of Critical Care Medicine and the World Federation of Pediatric Intensive and Critical Care Societies. 2015;16:264-9. doi:10.1097/pcc.0000000000000322.

47. Wong TE, Nguyen T, Shah SS, Brogan TV, Witmer CM. Antithrombin Concentrate Use in Pediatric Extracorporeal Membrane Oxygenation: A Multicenter Cohort Study. Pediatric critical care medicine : a journal of the Society of Critical Care Medicine and the World Federation of Pediatric Intensive and Critical Care Societies. 2016;17:1170-78. doi:10.1097/pcc.0000000000000955.

48. Mitchell L, Andrew M, Hanna K, Abshire T, Halton J, Wu J, et al. Trend to efficacy and safety using antithrombin concentrate in prevention of thrombosis in children receiving l-asparaginase for acute lymphoblastic leukemia. Results of the PAARKA study. Thrombosis and haemostasis. 2003;90:235-44. doi:10.1160/th02-11-0283.

49. Mccrindle BW, Manlhiot C, Holtby HM, Chan AK, Brandao LR, Rolland M, et al. Abstract 18061: Supplementation to Treat Antithrombin Deficiency Improves Sensitivity to Heparin, Anticoagulation and Decreased Thrombogenecity in Neonates and Infants Undergoing Cardiac Surgery With Cardiopulmonary Bypass. 2015;132:-.

50. Estepp JH, Smeltzer M, Reiss UM. The impact of quality and duration of enoxaparin therapy on recurrent venous thrombosis in children. Pediatric blood & cancer. 2012;59:105-9. doi:10.1002/pbc.23396.

51. Ross CE, Shih JA, Kleinman ME, Donnino MW. Pediatric Massive and Submassive Pulmonary Embolism: A Single-Center Experience. Hospital pediatrics. 2020;10:272-76. doi:10.1542/hpeds.2019-0290.

52. Ross C, Kumar R, Pelland-Marcotte MC, Mehta S, Kleinman ME, Thiagarajan RR, et al. Acute Management of High-Risk and Intermediate-Risk Pulmonary Embolism in Children: A Review. Chest. 2022;161:791-802. doi:10.1016/j.chest.2021.09.019.

53. Goldenberg NA, Durham JD, Knapp-Clevenger R, Manco-Johnson MJ. A thrombolytic regimen for high-risk deep venous thrombosis may substantially reduce the risk of postthrombotic syndrome in children. Blood. 2007;110:45-53. doi:10.1182/blood-2006-12-061234.

54. Levy M, Benson LN, Burrows PE, Bentur Y, Strong DK, Smith J, et al. Tissue plasminogen activator for the treatment of thromboembolism in infants and children. The Journal of pediatrics. 1991;118:467-72. doi:10.1016/s0022-3476(05)82170-5.

55. Farnoux C, Camard O, Pinquier D, Hurtaud-Roux MF, Sebag G, Schlegel N, et al. Recombinant tissue-type plasminogen activator therapy of thrombosis in 16 neonates. The Journal of pediatrics. 1998;133:137-40. doi:10.1016/s0022-3476(98)70193-3.

56. Manco-Johnson MJ, Nuss R, Hays T, Krupski W, Drose J, Manco-Johnson ML. Combined thrombolytic and anticoagulant therapy for venous thrombosis in children. The Journal of pediatrics. 2000;136:446-53. doi:10.1016/s0022-3476(00)90006-4.

57. Gupta AA, Leaker M, Andrew M, Massicotte P, Liu L, Benson LN, et al. Safety and outcomes of thrombolysis with tissue plasminogen activator for treatment of intravascular thrombosis in children. The Journal of pediatrics. 2001;139:682-8. doi:10.1067/mpd.2001.118428.

58. Knöfler R, Dinger J, Kabus M, Müller D, Lauterbach I, Rupprecht E, et al. Thrombolytic therapy in children--clinical experiences with recombinant tissue-plasminogen activator. Seminars in thrombosis and hemostasis. 2001;27:169-74. doi:10.1055/s-2001-14077.

59. Newall F, Browne M, Savoia H, Campbell J, Barnes C, Monagle P. Assessing the outcome of systemic tissue plasminogen activator for the management of venous and arterial thrombosis in pediatrics. Journal of pediatric hematology/oncology. 2007;29:269-73. doi:10.1097/MPH.0b013e318047b78b.

60. Leary SE, Harrod VL, de Alarcon PA, Reiss UM. Low-dose systemic thrombolytic therapy for deep vein thrombosis in pediatric patients. Journal of pediatric hematology/oncology. 2010;32:97-102. doi:10.1097/MPH.0b013e3181cc826e.

61. Goldenberg NA, Branchford B, Wang M, Ray C, Jr., Durham JD, Manco-Johnson MJ. Percutaneous mechanical and pharmacomechanical thrombolysis for occlusive deep vein thrombosis of the proximal limb in adolescent subjects: findings from an institution-based prospective inception cohort study of pediatric venous thromboembolism. Journal of vascular and interventional radiology : JVIR. 2011;22:121-32. doi:10.1016/j.jvir.2010.10.013.

62. Darbari DS, Desai D, Arnaldez F, Desai K, Kallen J, Strouse J, et al. Safety and efficacy of catheter directed thrombolysis in children with deep venous thrombosis. British journal of haematology. 2012;159:376-8. doi:10.1111/bjh.12025.

63. Dandoy CE, Kukreja KU, Gruppo RA, Patel MN, Tarango C. Outcomes in children with deep vein thrombosis managed with percutaneous endovascular thrombolysis. Pediatric radiology. 2015;45:719-26. doi:10.1007/s00247-014-3209-4.

64. Gaballah M, Shi J, Kukreja K, Raffini L, Tarango C, Keller M, et al. Endovascular Thrombolysis in the Management of Iliofemoral Thrombosis in Children: A Multi-Institutional Experience. Journal of vascular and interventional radiology : JVIR. 2016;27:524-30. doi:10.1016/j.jvir.2015.12.753.

65. Ansah DA, Patel KN, Montegna L, Nicholson GT, Ehrlich AC, Petit CJ. Tissue Plasminogen Activator Use in Children: Bleeding Complications and Thrombus Resolution. The Journal of pediatrics. 2016;171:67-72.e1-2. doi:10.1016/j.jpeds.2015.11.020.

66. Zengin E, Sarper N, Yazal Erdem A, Odaman Al I, Sezgin Evim M, Yaralı N, et al. Thrombolysis with Systemic Recombinant Tissue Plasminogen Activator in Children: A Multicenter Retrospective Study. Turkish journal of haematology : official journal of Turkish Society of Haematology. 2021;38:294-305. doi:10.4274/tjh.galenos.2021.2021.0038.

67. Belsky J, Warren P, Stanek J, Kumar R. Catheter-directed thrombolysis for submassive pulmonary embolism in children: A case series. Pediatric blood & cancer. 2020;67:e28144. doi:10.1002/pbc.28144.

68. Ji D, Gill AE, Durrence WW, Shah JH, Paden ML, Patel KN, et al. Catheter-Directed Pharmacologic Thrombolysis for Acute Submassive and Massive Pulmonary Emboli in Children and Adolescents-An Exploratory Report. Pediatric critical care medicine : a journal of the Society of Critical Care Medicine and the World Federation of Pediatric Intensive and Critical Care Societies. 2020;21:e15-e22. doi:10.1097/pcc.0000000000002172.

69. Engelberger RP, Kucher N. Ultrasound-assisted thrombolysis for acute pulmonary embolism: a systematic review. European heart journal. 2014;35:758-64. doi:10.1093/eurheartj/ehu029.

70. Kuo WT, Banerjee A, Kim PS, DeMarco FJ, Jr., Levy JR, Facchini FR, et al. Pulmonary Embolism Response to Fragmentation, Embolectomy, and Catheter Thrombolysis (PERFECT): Initial Results From a Prospective Multicenter Registry. Chest. 2015;148:667-73. doi:10.1378/chest.15-0119.

71. Piazza G, Hohlfelder B, Jaff MR, Ouriel K, Engelhardt TC, Sterling KM, et al. A Prospective, Single-Arm, Multicenter Trial of Ultrasound-Facilitated, Catheter-Directed, Low-Dose Fibrinolysis for Acute Massive and Submassive Pulmonary Embolism: The SEATTLE II Study. JACC Cardiovascular interventions. 2015;8:1382-92. doi:10.1016/j.jcin.2015.04.020.

72. Bloomer TL, El-Hayek GE, McDaniel MC, Sandvall BC, Liberman HA, Devireddy CM, et al. Safety of catheter-directed thrombolysis for massive and submassive pulmonary embolism: Results of a multicenter registry and meta-analysis. Catheterization and cardiovascular interventions : official journal of the Society for Cardiac Angiography & Interventions. 2017;89:754-60. doi:10.1002/ccd.26900.

73. Kucher N, Boekstegers P, Müller OJ, Kupatt C, Beyer-Westendorf J, Heitzer T, et al. Randomized, controlled trial of ultrasound-assisted catheter-directed thrombolysis for acute intermediate-risk pulmonary embolism. Circulation. 2014;129:479-86. doi:10.1161/circulationaha.113.005544.

74. Gamillscheg A, Nürnberg JH, Alexi-Meskishvili V, Werner H, Abdul-Kaliq H, Uhlemann F, et al. Surgical emergency embolectomy for the treatment of fulminant pulmonary embolism in a preterm infant. Journal of pediatric surgery. 1997;32:1516-8. doi:10.1016/s0022-3468(97)90581-9.

75. Maraj S, Bajpai E, Crespo G, Doshi K, Langan N, Jacobs LE, et al. Undefined hypercoaguable state associated with massive right ventricular thrombus and embolism in a previously healthy 17-year-old male. Echocardiography. 2003;20:439-42. doi:10.1046/j.1540-8175.2003.03075.x.

76. Cooper L, Moore C, Branchford B, Greffe B, Capocelli K, Kuder A, et al. Successful pulmonary artery embolectomy in a patient with a saddle Wilms tumor embolus. Pediatric blood & cancer. 2012;58:806-9. doi:10.1002/pbc.23215.

77. Lee SY, Baek JS, Kim GB, Kwon BS, Bae EJ, Noh CI, et al. Clinical significance of thrombosis in an intracardiac blind pouch after a Fontan operation. Pediatr Cardiol. 2012;33:42-8. doi:10.1007/s00246-011-0074-x.

78. Forbes TJ, Rosenthal GL, Reul GR, Jr., Ott DA, Feltes TF. Risk factors for life-threatening cavopulmonary thrombosis in patients undergoing bidirectional superior cavopulmonary shunt: an exploratory study. Am Heart J. 1997;134:865-71. doi:10.1016/s0002-8703(97)80009-9.

79. Dang MP, Cheng A, Garcia J, Lee Y, Parikh M, McMichael ABV, et al. Bringing PERT to Pediatrics: Initial Experience and Outcomes of a Pediatric Multidisciplinary Pulmonary Embolism Response Team (PERT). Chest. 2025;167:851-62. doi:10.1016/j.chest.2024.09.028.

80. Rosovsky R, Chang Y, Rosenfield K, Channick R, Jaff MR, Weinberg I, et al. Changes in treatment and outcomes after creation of a pulmonary embolism response team (PERT), a 10-year analysis. Journal of thrombosis and thrombolysis. 2019;47:31-40. doi:10.1007/s11239-018-1737-8.

81. Jen WY, Kristanto W, Teo L, Phua J, Yip HS, MacLaren G, et al. Assessing the Impact of a Pulmonary Embolism Response Team and Treatment Protocol on Patients Presenting With Acute Pulmonary Embolism. Heart, lung & circulation. 2020;29:345-53. doi:10.1016/j.hlc.2019.02.190.

82. Wright C, Elbadawi A, Chen YL, Patel D, Mazzillo J, Acquisto N, et al. The impact of a pulmonary embolism response team on the efficiency of patient care in the emergency department. Journal of thrombosis and thrombolysis. 2019;48:331-35. doi:10.1007/s11239-019-01875-0.

83. Xenos ES, Davis GA, He Q, Green A, Smyth SS. The implementation of a pulmonary embolism response team in the management of intermediate- or high-risk pulmonary embolism. Journal of vascular surgery Venous and lymphatic disorders. 2019;7:493-500. doi:10.1016/j.jvsv.2018.11.014.

84. Chaudhury P, Gadre SK, Schneider E, Renapurkar RD, Gomes M, Haddadin I, et al. Impact of Multidisciplinary Pulmonary Embolism Response Team Availability on Management and Outcomes. The American journal of cardiology. 2019;124:1465-69. doi:10.1016/j.amjcard.2019.07.043.

85. Shaoxiu S, Yongsheng X. Clinical analysis of 9 cases of mycoplasma pneumoniae pneumonia complicated with pulmonary embolism in children and literature review. Journal of Shandong University. 2023;61:96-103.

86. Sheng CQ, Yang CF, Ao Y, Zhao ZY, Li YMJE, Medicine T. Mycoplasma pneumoniae pneumonia with pulmonary embolism: A study on pediatric cases in Jilin province of China. 2021.

87. Fu Y, Zhang TQ, Dong CJ, Xu YS, Dong HQ, Ning J. Clinical characteristics of 14 pediatric mycoplasma pneumoniae pneumonia associated thrombosis: a retrospective study. BMC cardiovascular disorders. 2023;23:1. doi:10.1186/s12872-022-03030-9.

88. Han C, Zhang T, Zheng J, Jin P, Zhang Q, Guo W, et al. Analysis of the risk factors and clinical features of Mycoplasma pneumoniae pneumonia with embolism in children: a retrospective study. Italian journal of pediatrics. 2022;48:153. doi:10.1186/s13052-022-01344-0.

89. Reed RA, Teitelbaum GP, Stanley P, Mazer MJ, Tonkin IL, Rollins NK. The use of inferior vena cava filters in pediatric patients for pulmonary embolus prophylaxis. Cardiovascular and interventional radiology. 1996;19:401-5. doi:10.1007/bf02577627.

90. Cahn MD, Rohrer MJ, Martella MB, Cutler BS. Long-term follow-up of Greenfield inferior vena cava filter placement in children. Journal of vascular surgery. 2001;34:820-5. doi:10.1067/mva.2001.118801.

91. Raffini L, Cahill AM, Hellinger J, Manno C. A prospective observational study of IVC filters in pediatric patients. Pediatric blood & cancer. 2008;51:517-20. doi:10.1002/pbc.21622.

92. Kukreja KU, Gollamudi J, Patel MN, Johnson ND, Racadio JM. Inferior vena cava filters in children: our experience and suggested guidelines. Journal of pediatric hematology/oncology. 2011;33:334-8. doi:10.1097/MPH.0b013e3182191dac.

93. Blevins EM, Glanz K, Huang YS, Raffini L, Shinohara RT, Witmer C. A Multicenter Cohort Study of Inferior Vena Cava Filter Use in Children. Pediatric blood & cancer. 2015;62:2089-93. doi:10.1002/pbc.25662.

94. Rottenstreich A, Revel-Vilk S, Bloom AI, Kalish Y. Inferior vena cava (IVC) filters in children: A 10-year single center experience. Pediatric blood & cancer. 2015;62:1974-8. doi:10.1002/pbc.25641.

95. Guzman AK, Zahra M, Trerotola SO, Raffini LJ, Itkin M, Keller MS, et al. IVC filter retrieval in adolescents: experience in a tertiary pediatric center. Pediatric radiology. 2016;46:534-40. doi:10.1007/s00247-015-3519-1.

96. Lv YL, Guan N, Ding J, Yao Y, Xiao HJ, Zhong XH, et al. Spectrum of thrombotic complications and their outcomes in Chinese children with primary nephrotic syndrome. Italian journal of pediatrics. 2020;46:182. doi:10.1186/s13052-020-00942-0.

97. Suri D, Ahluwalia J, Saxena AK, Sodhi KS, Singh P, Mittal BR, et al. Thromboembolic complications in childhood nephrotic syndrome: a clinical profile. Clinical and experimental nephrology. 2014;18:803-13. doi:10.1007/s10157-013-0917-2.

.

Appendix 3 Search strategies

PubMed:

(lung embolism*[Title/Abstract]) OR (lung thromboembolism*[Title/Abstract]) OR (pulmonary embolism*[Title/Abstract]) OR (pulmonary thromboembolism*[Title/Abstract]) OR ([Pulmonary Infarction](https://www.ncbi.nlm.nih.gov/mesh/68054060)*[Title/Abstract]) OR (Pulmonary Infarct*[Title/Abstract]) OR ("Pulmonary Embolism"[Mesh]) OR ("Pulmonary Infarction"[Mesh]) Filters: Child: birth-18 years

Embase:

#1 exp lung embolism/ or lung embolism*.mp.

#2 lung thromboembolism*.mp.

#3 pulmonary embolism*.mp.

#4 pulmonary thromboembolism*.mp.

#5 exp lung infarction/ or Pulmonary Infarction*.mp.

#6 Pulmonary Infarct*.mp.

#7 1 or 2 or 3 or 4 or 5 or 6

#8 limit 7 to (infant <to one year> or child <unspecified age> or preschool child <1 to 6 years> or school child <7 to 12 years> or adolescent <13 to 17 years>)

Cochrane:

#1 ("lung embolism*"):ti,ab,kw

#2 ("lung thromboembolism*"):ti,ab,kw

#3 ("pulmonary embolism*"):ti,ab,kw

#4 ("pulmonary thromboembolism*"):ti,ab,kw

#5 ("Pulmonary Infarction*"):ti,ab,kw

#6 ("Pulmonary Infarct*"):ti,ab,kw

#7 MeSH descriptor: [Pulmonary Embolism] explode all trees

#8 MeSH descriptor: [Pulmonary Infarction] explode all trees

#9 #1 or #2 or #3 or #4 or #5 or #6 or #7 or #8

#10 (children):ti,ab,kw

#11 (child):ti,ab,kw

#12 (pediatric*):ti,ab,kw

#13 (teenager*):ti,ab,kw

#14 (adolescent*):ti,ab,kw

#15 MeSH descriptor: [Infant] explode all trees

#16 MeSH descriptor: [Child] explode all trees

#17 MeSH descriptor: [Adolescent] explode all trees

#18 #10 or #11 or #12 or #13 or #14 or #15 or #16 or #17

#19 #9 and #18

中国知网：[(主题=肺栓塞 + 肺血栓 + 肺血管阻塞症 + 肺动脉血栓 + 肺静脉血栓 + 肺梗) AND (主题=儿科 + 儿童 + 小儿 + 患儿 + 婴儿 + 幼儿 + 青少年)](http://182.150.59.104:8888/https/77726476706e69737468656265737421fbf952d2243e635930068cb8/kns8/AdvSearch?id=445&dbcode=CFLS&searchtype=gradeSearch&ishistory=1" \t "http://182.150.59.104:8888/https/77726476706e69737468656265737421fbf952d2243e635930068cb8/kns8/manage/_blank" \o "(主题=肺栓塞 + 肺血栓 + 肺血管阻塞症 + 肺动脉血栓 + 肺静脉血栓 + 肺梗) AND (主题=儿科 + 儿童 + 小儿 + 患儿 + 婴儿 + 幼儿 + 青少年))

维普：

(M=肺栓塞 OR R=肺栓塞 OR M=肺血栓 OR R=肺血栓 OR M=肺血管阻塞症 OR R=肺血管阻塞症 OR M=肺动脉血栓 OR R=肺动脉血栓 OR M=肺静脉血栓 OR R=肺静脉血栓 OR M=肺梗 OR R=肺梗) AND (M=儿科 OR R=儿科 OR M=儿童 OR R=儿童 OR M=小儿 OR R=小儿 OR M=患儿 OR R=患儿 OR M=婴儿 OR R=婴儿 OR M=幼儿 OR R=幼儿 OR M=青少年 OR R=青少年)

万方：

(主题:("肺栓塞") or 主题:("肺血栓") or 主题:("肺血管阻塞症") or 主题:("肺动脉血栓") or 主题:("肺静脉血栓") or 主题:("肺梗")) and (主题:("儿科") or 主题:("儿童") or 主题:("小儿") or 主题:("患儿") or 主题:("婴儿") or 主题:("幼儿") or 主题:("青少年"))
